# Unsatisfied Reporting Quality of Clinical Trials Evaluating Immune Checkpoint Inhibitor Therapy in Cancer

**DOI:** 10.3389/fimmu.2021.736943

**Published:** 2021-10-05

**Authors:** Chen Chen, Yixin Zhou, Xuanye Zhang, Yuhong Wang, Li-na He, Zuan Lin, Tao Chen, Yongluo Jiang, Shaodong Hong, Li Zhang

**Affiliations:** ^1^ Department of Radiation Oncology, Sun Yat-Sen University Cancer Center, Guangzhou, China; ^2^ State Key Laboratory of Oncology in South China, Collaborative Innovation Center for Cancer Medicine, Guangdong Key Laboratory of Nasopharyngeal Carcinoma Diagnosis and Therapy, Sun Yat-Sen University Cancer Center, Guangzhou, China; ^3^ Department of Very Important Person (VIP) Region, Sun Yat-Sen University Cancer Center, Guangzhou, China; ^4^ Department of Medical Oncology, Sun Yat-Sen University Cancer Center, Guangzhou, China; ^5^ Department of Endoscopy, Sun Yat-Sen University Cancer Center, Guangzhou, China; ^6^ Department of Clinical Research, Sun Yat-Sen University Cancer Center, Guangzhou, China; ^7^ Department of Nuclear Medicine, Sun Yat-Sen University Cancer Center, Guangzhou, China

**Keywords:** reporting quality, clinical trials, immune checkpoint inhibitor, immune therapy, cancer, evaluating

## Abstract

**Background:**

More and more immune-oncology trials have been conducted for treating various cancers, yet it is unclear what the reporting quality of immune-oncology trials is,and characteristics associated with higher reporting quality.

**Objective:**

This study aims to evaluate the reporting quality of immune-oncology trials.

**Methods:**

The PubMed and Cochrane library were searched to identify all English publications of clinical trials assessing immunotherapy for cancer. Reporting quality of immune-oncology trials was evaluated by a quality score with 11 points derived from the Trial Reporting in Immuno-Oncology (TRIO) statement, which contained two parts: an efficacy score of 6 points and toxicity score of 5 point. Linear regression was used to identify characteristics associated with higher scores.

**Results:**

Of the 10,169 studies screened, 298 immune-oncology trial reports were enrolled. The mean quality score, efficacy score, and toxicity score were 6.46, 3.61, and 2.85, respectively. The most common well-reported items were response evaluation criteria (96.0%) and toxicity grade (98.7%), followed by Kaplan-Meier survival analyses (80.5%). Treatment details beyond progression (12.8%) and toxicity onset time and duration (7.7%) were poorly reported. Multivariate regression revealed that higher impact factor (IF) (IF >20 vs. IF <5, *p* < 0.001), specific tumor type (*p* = 0.018 for lung, *p* = 0.021 for urinary system, vs. pan cancer), and a certain kind of immune checkpoint blocking agent (*p* < 0.001 for anti-PD-1 or multiagents, vs. anti-CTLA-4) were independent predictors of higher-quality score. Similar independent predictive characteristics were revealed for high-efficacy score. Only IF >20 had a significant high-toxicity score (*p* < 0.001).

**Conclusion:**

Immune-oncology trial reports presented an unsatisfied quality score, especially in the reporting of treatment details beyond progression and toxicity onset time and duration. High IF journals have better reporting quality. Future improvement of trial reporting was warranted to the benefit-risk assessment of immunotherapy.

## Introduction

With the success of immune checkpoint blockade (ICB), immunotherapy has revolutionized cancer therapy. More and more immunotherapies have been approved for treating various cancers (1, 2). The immune checkpoint blocking agents for immunotherapies include anticytotoxic T-lymphocyte antigen 4 (CTLA-4, ipilimumab, tremelimumab), antiprogrammed cell death protein 1 (PD-1, nivolumab, pembrolizumab), and antiprogrammed death-ligand 1 (PD-L1, atezolizumab, avelumab, durvalumab) agents (3, 4).

Clinical trials are considered essential to advancing and evaluating the use of ICB in cancer treatment (5). Biomedical publications of various journals are key methods for disseminating the design, conduct, results, and conclusions of these trials. The published reports should provide the reader with the ability to fully understand the trial and make informed judgments of trial results. Thus, it needs to be a unified standard to ensure the quality of the reports.

The Consolidated Standards of Reporting Trials (CONSORT) statement provides guidance to authors regarding essential items that should be included in trial reports and can be also applied to immune-oncology (IO) trials (6, 7). However, distinct mechanisms of IO therapies exhibit unique efficacy and toxicity compared with traditional cancer treatments such as chemotherapy, which may lead to additional considerations for reporting guidelines of IO clinical trials (8, 9). Based on this fact, the Trial Reporting in Immuno-Oncology (TRIO) statement is developed by American Society of Clinical Oncology (ASCO) and the Society for Immunotherapy of Cancer (SITC) to improve the interpretation and comparison across IO trials (10, 11). There have been literatures evaluating the quality of randomized clinical trials (RCT) reports based on the CONSORT statement (12–14), but no studies specifically evaluate the report quality of IO trials. Therefore, the purpose of this study is to evaluate the reporting quality of IO trials based on the TRIO statement. In addition, we also investigated the publications’ characteristics associated with higher quality in IO trial reporting.

## Materials and Methods

### Trial Selection

We searched PubMed (https://pubmed.ncbi.nlm.nih.gov) and Cochrane (https://www.cochranelibrary.com) to identify all English publications of clinical trials assessing immunotherapy for cancer. The search was performed in September 16, 2019, using the keywords as follows: cancer (neoplasia, neoplasias, neoplasm, tumors, tumor, cancers, malignancy, malignancies, carcinoma, leukemia, lymphoma, melanoma, glioma); immune checkpoint inhibitor (immune checkpoint blocking agent, immune therapy, immunotherapy, immunotherapies, immuno-oncology treatment, anticytotoxic T-lymphocyte antigen 4, CTLA-4, ipilimumab, tremelimumab, antiprogrammed cell death protein 1, PD-1, nivolumab, pembrolizumab, antiprogrammed death-ligand 1, PD-L1, atezolizumab, avelumab, durvalumab); and clinical trials. Exclusion criteria included: non-article (review, editorial paper, abstract only, and conference paper), registration information and clinical trial protocols, non-clinical trials, pilot trials, post-hoc or pooled analysis of clinical trials, non-immunotherapy, and non-malignant tumor studies.

### Quantitative Scoring System for Quality of Trial Reporting in Immuno-Oncology

A trial reporting of immuno-oncology quality score (TRIOQS) based on TRIO statement was defined by two of the authors (CC and SH). The score was based on the recommendations of TRIO statement except the combination or sequencing of immunotherapies reporting standard (**Table 1**). This scoring system contained two parts, the one was efficacy score (TRIOQS-E, items 1–6 in **Table 1**) and the other one was toxicity score (TRIOQS-T, items 7–11 in **Table 1**). Each item enrolled in TRIOQS was scored as 1 if it was reported or 0 if it was not reported at all; each item was weighted with equal importance. For those recommendations with several subcomponents, a score of 1 was given if any one of them was reported. The 12th recommendation of TRIO statement was excluded because it was especially for clinical trials with combination or sequencing of immunotherapies.

**Table 1:** Quality of immune-oncology clinical trial reporting using items from the Trial Reporting in Immuno-Oncology (TRIO) statement.

TRIO statement No.	Descriptor of the TRIO standards	Descriptor of the reporting quality criteria	Score	Trials in which item was adequately reported	
				No.	%
Efficacy reporting standards					
1	Report the criteria used to evaluate response to therapy and the rationale for the chosen criteria.	Response evaluation criteria were reported in the main text or appendix.	1	286	96.0
		- With rationale for the chosen criteria	–	5	1.7
		- Without rationale for the chosen criteria	–	281	94.3
2	Include spider plots or swimmer plots in efficacy descriptions to better report kinetics of response.	Spider plots or swimmer plots were presented to report response in the main text or appendix.	1	140	47.0
3	Report how disease control rate is defined and how its components are assessed.	Nonstandardized end points (disease control, objective response, clinical benefit) were defined in the main text or appendix.	1	223	74.8
		- With components assessed	–	220	73.8
		- Without components assessed	–	3	1.0
4	Report criteria that allow patients to continue treatment beyond disease progression.	Criteria for treating beyond progression were reported in the main text or appendix.	1	149	50.0
5	Report the number (proportion) of patients who are treated beyond progression, treatment beyond progression duration, emergence of new toxicity, and efficacy after initial progression.	Details of treatment beyond progression were reported in the main text or appendix, including number (proportion) of patients, or treatment duration, or new toxicity, or efficacy.	1	38	12.8
		- Number (proportion) of patients	–	38	12.8
		- Treatment duration	–	6	2.0
		- New toxicity	–	4	1.3
		- Efficacy	–	15	5.0
6	Report progression-free survival and overall survival using Kaplan-Meier analyses.	Progression-free survival or overall survival was reported by Kaplan-Meier analyses in the main text or appendix.	1	240	80.5
		- Progression-free survival	–	200	67.1
		- Overall survival	–	216	72.5
Toxicity reporting standards					
7	Differentiate between the clinical diagnoses of IO toxicity and the specific symptoms that led to the diagnoses.	Clinical diagnoses of IO toxicity and the associated symptoms were both reported in the main text or appendix.	1	181	60.7
8	If the prespecified clinical diagnoses used in data collection belong to categories such as “immune-related adverse events” or “adverse events of special interest,” report how these terms are defined and why these categories were selected for trial reporting.	The terms “immune-related adverse events” (irAE) or “adverse events of special interest” (AEOSI) were defined in the main text or appendix.	1	158	53.0
		- With rationale for the selection	–	5	1.7
		- Without rationale for the selection	–	153	51.3
9	Report all toxicity by specific grade.	Toxicities were reported in all grades or in highlight grades 3 to 4 according to specific criteria such as CTCAE in the man text or appendix.	1	294	98.7
10	Report clinical interventions used to manage IO toxicity.	Clinical management of adverse events were reported in the main text or appendix, such as dose delays and use of immunosuppression.	1	192	64.4
11	Report time of onset and duration of IO toxicity.	The time of toxicity onset and of toxicity resolution were reported in the main text or appendix.	1	23	7.7
		- Onset time	–	18	6.0
		- Duration time	–	19	6.4
Combination or sequencing of immunotherapies reporting standard					
12	Report the scientific hypothesis for the combination or sequence on the basis of preclinical and/or clinical data as well as the rationale for the selection of the particular dose(s) and sequence of agents.	This item was not evaluated, because it is especially for clinical trials with combination or sequencing of immunotherapies.	NA	NA	NA

NA, not applicable.

The scoring system was piloted on 10 randomly selected publications (110 items) by two authors (CC and YZ) who were blinded to each other’s evaluation results. Among 110 items, 6 discrepancies were identified, and all were successfully resolved by consensus. Based on this consensus, the two authors (CC and YZ) evaluated the remaining publications.

### Trial Characteristic Selection and Definition

Several trial characteristics that could affect the quality score were selected. Year of publication was directly extracted as continuous variable. Journal impact factor was referred to 2018 and classified as four groups: <5, 5 to 10, 10 to 20, and >20. Trial phase was also concerned and consisted of phase I (I, or I/II), phase II (II, or II/III), and phase III (III or III/IV). Trials were considered as industry funded if they received any form of industry funding. Number of participating centers was calculated as three groups according to the median: 1 to 12, 13 to 246, and unknown group. Intercontinental trials were that recruited patients from more than one continent. Nonintercontinental trials were conducted in north America only, other regions (Asia only, Europe only, Oceania only), and unknown regions. The tumor types included in trials could be divided into the following four categories: pan cancer, lung cancer, melanoma, urinary system cancer, and other cancers. Based on the mechanism, immune checkpoint blocking agent in immunotherapy contained anti-CTLA-4, anti-PD-1, anti-PD-L1, and any mix of the above. According to the treatment strategy, immunotherapy could be used alone or combined with other therapy.

### Statistical Analysis

The TRIOQS was calculated as the sum of the score of items in **Table 1** and expressed as an integer from 0 to 11. TRIOQS scores were descripted using mean and standard error (SE). Single-item frequencies were compared between subgroups by Chi-square tests.

Univariate and multivariate linear regression analyses were used to identify trial characteristics associated with higher TRIOQS. Given that it was deemed desirable to include as many characteristics associated with reporting quality as possible, the multivariable regression included all mentioned covariates. Violin plots were used to visually show the significant differences in TRIOQS among subgroups of statistically characteristics. Statistical analyses were performed using R software (http://www.R-project.org/). All tests were two-tailed, with *p* < 0.05 considered statistically significant.

## Results

### Characteristics of Trials

**Figure 1:**
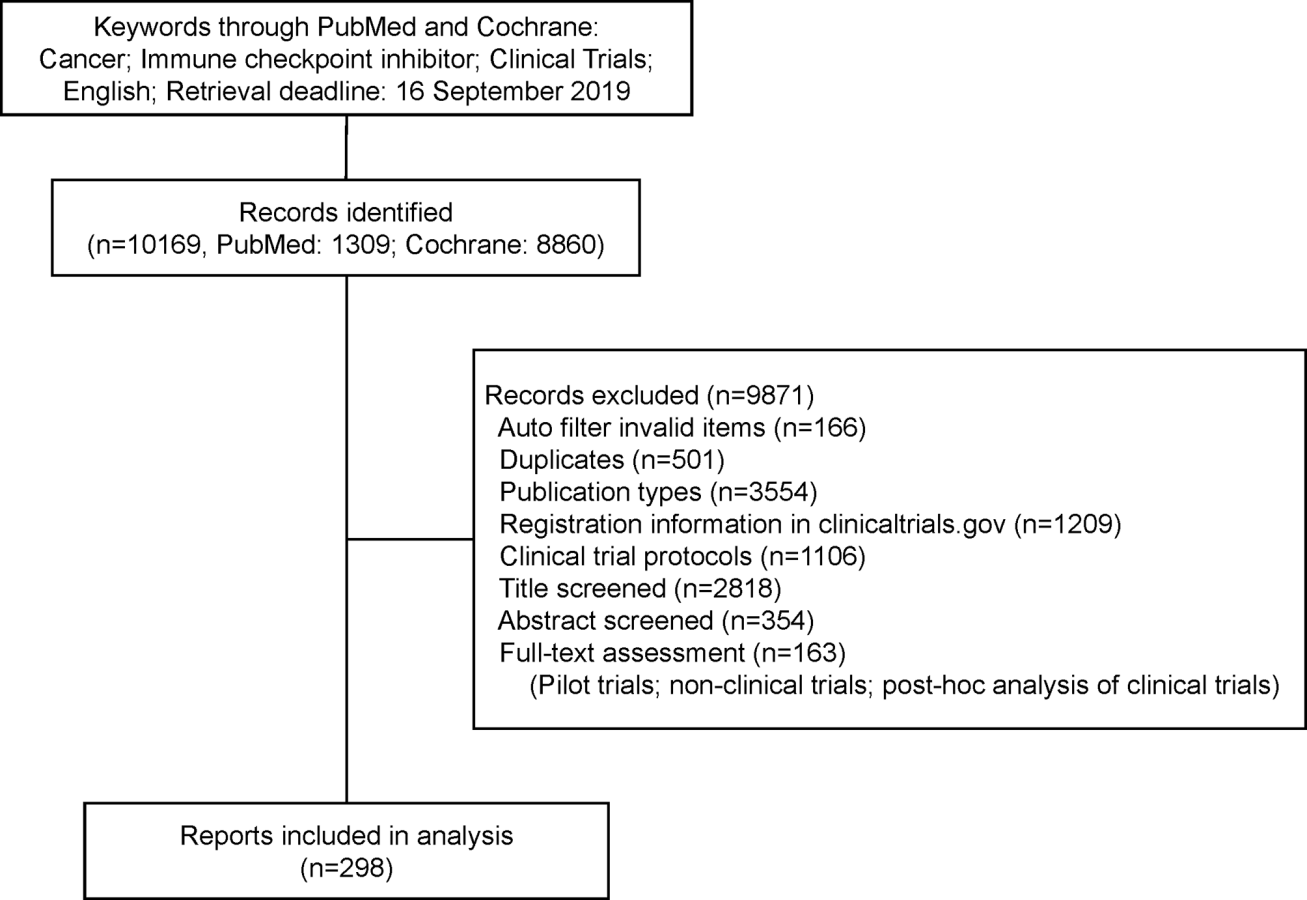
Flowchart. Identifying reports on immuno-oncology clinical trial.

From the 10,169 studies initially screened, a total of 298 immuno-oncology clinical trial reports were included in the present analysis (**Figure 1**; the list in the **Supplementary Materials**). Trials’ characteristics are listed in **Table 2**. The number of published trials almost monotonously increased with the year. More than half trials (*n* = 173, 58%) were published in journals with IF >20, including Lancet Oncology (*n* = 50, 16.8%), Journal of Clinical Oncology (*n* = 45, 15.1%), and The New England Journal of Medicine (*n* = 38, 12.8%). Only 45 trials were single center, and 10 trials enrolled more than 200 centers. The main treatment strategy was immunotherapy alone (*n* = 180, 60.4%). With the latest of publication year, the proportion of immunotherapy combined with other therapy increased (2009–2015: 23/69, 33.3%; 2016: 17/48, 35.4%; 2017: 18/68, 26.5%; 2018: 37/69, 53.6%; 2019: 23/44, 52.3%; *p* = 0.005), and the proportion of anti-PD-1 agent also increased (2009–2015: 21/69, 30.4%; 2016: 25/48, 52.1%; 2017: 38/68, 55.9%; 2018: 31/69, 44.9%; 2019: 26/44, 59.1%; *p* = 0.012).

**Table 2:** Trial characteristics.

Characteristic	Trials (*N* = 298)	
	No.	%
Year of publication		
2009	3	1.0
2010	9	3.0
2011	3	1.0
2012	8	2.7
2013	9	3.0
2014	9	3.0
2015	28	9.4
2016	48	16.1
2017	68	22.8
2018	69	23.2
2019	44	14.8
Journal impact factor		
<5	47	15.8
5–10	44	14.8
10–20	34	11.4
>20	173	58.0
Trial phase		
I	124	41.6
II	104	34.9
III	70	23.5
Source of trial funding		
No industry funding	31	10.4
Industry funding	267	89.6
Center number		
Median	12
Interquartile range	4–49
1–12	139	46.6
13–246	114	38.3
Unknown	45	15.1
Region in which trial was conducted		
Intercontinential	150	50.3
North America	82	27.5
Others	50	16.8
Unknown	16	5.4
Tumor type		
Pan cancer	22	7.4
Lung	65	21.8
Melanoma	79	26.5
Urinary system	39	13.1
Others	93	31.2
Immunotherapy strategy		
Alone	180	60.4
Combined with other therapy	118	39.6
- Chemotherapy	31	10.4
- Target therapy	31	10.4
- Radiotherapy	9	3.0
- Immunotherapy	26	8.7
- Ablation	0	0.0
- Surgery	4	1.3
- Others	20	6.7
Immune checkpoint blocking agent		
Anti-CTLA-4	76	25.5
Anti-PD-1	141	47.3
Anti-PD-L1	51	17.1
Multiagents	30	10.1

CTLA-4, cytotoxic T-lymphocyte antigen 4; PD-1, programmed cell death protein 1; PD-L1, programmed death-ligand 1.

### Quality Score According to TRIO Statement

The mean TRIOQS was 6.46 on an 11-point scale (range, 1 to 11; 95% CI, 6.23 to 6.69). Two hundred thirty-eight trials (79.9%) got a score of 5 to 9, while 27 trials (9.1%) have a score ≤3. Only two trials were found with a score of 11. The mean TRIOQS-E was 3.61 on a 6-point scale (range, 0–6; 95% CI, 3.45 to 3.77), with three trials having a score of 0 and 22 trials having a score of 6. The mean TRIOQS-T was 2.85 on a 5-point scale (range, 0–5; 95% CI, 2.72 to 2.98), with four trials having a score of 0 and 14 trials having a score of 5.

The most common well-reported items were response evaluation criteria (item 1, 96.0%) and toxicity grade (item 9, 98.7%), followed by Kaplan-Meier survival analyses (item 6, 80.5%). Spider or swimmer plots were presented more frequently by phase I trials (*n* = 74 of 124 trials, 59.7%) than by phase II (*n* = 47 of 104 trials, 45.2%) and phase III trials (*n* = 19 of 70 trials, 27.1%; *p* < 0.001). Criteria for continuous treatment beyond progression and definition of new adverse event terms (irAE or AEOSI), which were unique to immune-oncology therapy, were clearly described in 50% and 53.0% trials separately. However, treatment details beyond progression (item 5, 12.8%) and toxicity onset time and duration (item11, 7.7%) were poorly reported. It was worth noting that the reasons for the criteria selection were not fully explained in almost all trials.

### Characteristics Associated With Reporting Quality

The results of univariable and multivariable linear regressions are listed in **Table 3**. Although all characteristics were statistically significant in univariable analysis, multivariate regression only revealed that higher IF (IF >20 vs. IF <5, *p* < 0.001), specific tumor type (lung vs. pan cancer, *p* = 0.018; urinary system vs. pan cancer, *p* = 0.021), and a certain kind of immune checkpoint blocking agent (anti-PD-1 vs. anti-CTLA-4, *p* < 0.001; multiagents vs. anti-CTLA-4, *p* < 0.001) were independent predictors of higher TRIOQS.

**Table 3:** Results of linear regression analyses of trial characteristics predicting TRIOQS (0-11 scale).

Trial characteristic	TRIOQS		Linear regression					
			Univariate analysis			Multivariate analysis		
	Mean	SE	Estimate	SE	*p*	Estimate	SE	*p*
Year of publication, continuous	–		0.12	0.05	0.028	−0.00070	0.055	0.990
Journal impact factor								
<5	5.40	1.9	Reference		<0.001	Reference		
5–10	5.00	1.58	−0.40	0.36		−0.11	0.35	0.744
10–20	5.06	1.79	−0.35	0.39		−0.96	0.38	0.013
>20	7.39	1.67	1.98	0.28		1.15	0.31	<0.001
Trial phase								
I	6.04	2.06	Reference		<0.001	Reference		
II	6.41	2.08	0.37	0.26		0.18	0.24	0.452
III	7.26	1.66	1.22	0.30		0.26	0.28	0.362
Source of trial funding								
No industry funding	5.19	1.99	Reference		<0.001	Reference		
Industry funding	6.60	1.98	1.41	0.38		0.46	0.34	0.172
Center number								
1–12	5.71	1.92	Reference		<0.001	Reference		
13–246	7.27	1.92	1.56	0.24		0.15	0.29	0.603
Unknown	6.69	1.81	0.98	0.33		0.15	0.37	0.679
Region in which trial was conducted								
Intercontinential	7.22	1.85	Reference		<0.001	Reference		
North America	5.73	2.01	−1.49	0.26		−0.34	0.31	0.281
Others	5.48	1.88	−1.74	0.31		−0.56	0.34	0.100
Unknown	6.06	1.44	−1.16	0.49		−0.75	0.52	0.149
Tumor type								
Pan cancer	4.82	1.79	Reference		<0.001	Reference		
Lung	6.86	1.88	2.04	0.49		1.08	0.46	0.018
Melanoma	6.20	1.99	1.38	0.47		0.81	0.46	0.076
Urinary system	7.05	2.16	2.23	0.52		1.12	0.48	0.021
Others	6.53	1.96	1.71	0.47		0.79	0.43	0.068
Immunotherapy strategy								
Alone	6.76	1.90	Reference		0.002	Reference		
Combined with other therapy	6.00	2.14	−0.76	0.24		−0.28	0.24	0.245
Immune checkpoint blocking agent								
Anti-CTLA-4	5.09	1.71	Reference		<0.001	Reference		
Anti-PD-1	6.91	2.02	1.82	0.26		1.17	0.34	<0.001
Anti-PD-L1	6.57	1.85	1.48	0.34		0.59	0.41	0.152
Multiagents	7.60	1.30	2.51	0.40		1.50	0.41	<0.001

TRIOQS, trial reporting of immuno-oncology quality score; CTLA-4, cytotoxic T-lymphocyte antigen 4; PD-1, programmed cell death protein 1; PD-L1, programmed death-ligand 1.

**Figure 2:**
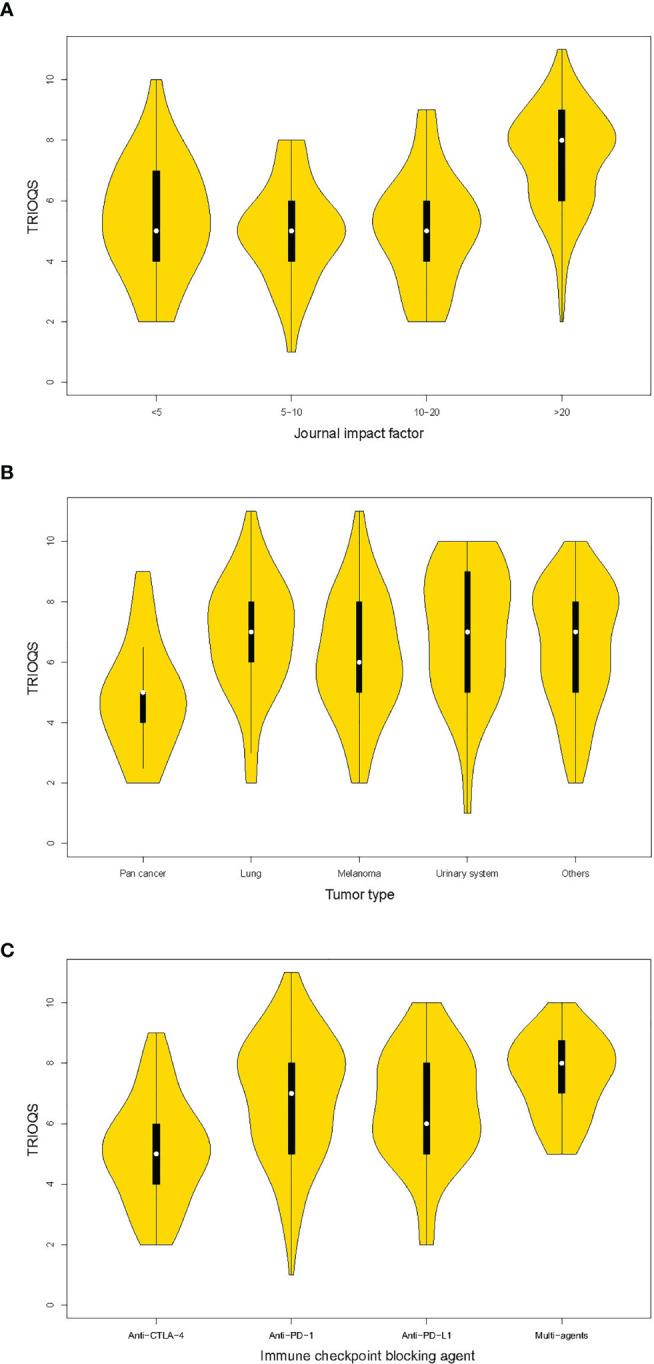
Violin plots of trial reporting of immuno-oncology quality score (TRIOQS) by characteristics of publications. **(A)** Journal impact factor; **(B)** tumor type; **(C)** immune checkpoint blocking agent. The white dot is the median value. The black box ranges from the lower quartile to the upper quartile values, and the thin black line indicates 95% confidence interval. The outer shape is the kernel density estimation.

Specifically, articles with IF >20 had a TRIOQS on average 1.15 points higher than those with IF <5 (**Figure 2A**). Publications of lung cancer and urinary system cancer had a TRIOQS that was 1.08 and 1.12 point higher than those of pan cancers separately. While publications of melanoma and other cancers respectively had a TRIOQS that was 0.81 and 0.79 point potential higher than those of pan cancers (**Figure 2B**). The TRIOQS of trials on anti-PD-1 agent was higher than those on anti-CTLA-4 agent by a mean of 1.17 points and trials on multiagents had an average 1.5 points higher TRIOQS than those on anti-CTLA-4 agent (**Figure 2C**).

Similar independent predictive characteristics were revealed for high TRIOQS-E in multivariable regression, including IF >20 (*vs*. IF <5, *p* = 0.034), phase II (*vs*. phase I, *p* = 0.021), specific cancer (lung cancer *vs*. pan cancer, *p* = 0.030; urinary system cancer vs. pan cancer, *p* = 0.013; other cancers *vs*. pan cancers, *p* = 0.012), certain kinds of immune checkpoint blocking agent (anti-PD-1, anti-PD-L1, and multiagents vs. anti-CTLA-4, all *p* < 0.001) (**Table 4**). However, only IF >20 had a significant high TRIOQS-T (*p* < 0.001) (**Table 5**).

**Table 4:** Results of linear regression analyses of trial characteristics predicting TRIOQS-E (0–6 scale).

Trial characteristic	TRIOQS-E		Linear regression					
			Univariate analysis			Multivariate analysis		
	Mean	SE	Estimate	SE	*p*	Estimate	SE	*p*
Year of publication, continuous	–		0.13	0.03	<0.001	0.01	0.04	0.704
Journal impact factor								
<5	3.09	1.44	Reference		<0.001	Reference		
5–10	2.82	1.26	−0.27	0.26		0.03	0.24	0.910
10–20	2.85	1.31	−0.23	0.28		−0.73	0.27	0.007
>20	4.10	1.18	1.02	0.21		0.46	0.22	0.034
Trial phase								
I	3.36	1.47	Reference		0.021	Reference		
II	3.71	1.36	0.35	0.18		0.39	0.17	0.021
III	3.90	1.16	0.54	0.20		0.06	0.20	0.763
Source of trial funding								
No industry funding	2.81	1.25	Reference		<0.001	Reference		
Industry funding	3.70	1.36	0.90	0.26		0.35	0.23	0.135
Center number								
1–12	3.21	1.35	Reference		<0.001	Reference		
13–246	3.99	1.30	0.78	0.17		−0.14	0.20	0.50
Unknown	3.89	1.32	0.68	0.23		0.15	0.25	0.56
Region in which trial was conducted								
Intercontinential	4.03	1.21	Reference		<0.001	Reference		
North America	3.16	1.37	−0.87	0.18		−0.24	0.22	0.270
Others	3.14	1.48	−0.89	0.21		−0.39	0.24	0.102
Unknown	3.50	1.37	−0.53	0.35		−0.46	0.36	0.200
Tumor type								
Pan cancer	2.64	1.29	Reference		<0.001	Reference		
Lung	3.82	1.16	1.18	0.33		0.69	0.32	0.030
Melanoma	3.16	1.23	0.53	0.32		0.44	0.32	0.166
Urinary system	4.00	1.67	1.36	0.35		0.83	0.33	0.013
Others	3.91	1.33	1.28	0.31		0.75	0.30	0.012
Immunotherapy strategy								
Alone	3.89	1.29	Reference		<0.001	Reference		
Combined with other therapy	3.19	1.41	−0.70	0.16		−0.26	0.17	0.126
Immune checkpoint blocking agent								
Anti-CTLA-4	2.47	1.09	Reference		<0.001	Reference		
Anti-PD-1	4.02	1.31	1.55	0.17		1.14	0.24	<0.001
Anti-PD-L1	3.88	1.13	1.41	0.22		1.00	0.29	<0.001
Multiagents	4.10	1.12	1.63	0.26		1.21	0.29	<0.001

TRIOQS-E, trial reporting of immuno-oncology quality score-efficacy score; CTLA-4, cytotoxic T-lymphocyte antigen 4; PD-1, programmed cell death protein 1; PD-L1, programmed death-ligand 1.

**Table 5:** Results of linear regression analyses of trial characteristics predicting TRIOQS-T (0–5 scale).

Trial characteristic	TRIOQS-T		Linear regression					
			Univariate analysis			Multivariate analysis		
	Mean	SE	Estimate	SE	*p*	Estimate	SE	*p*
Year of publication, continuous	–		−0.01	0.03	0.741	−0.02	0.04	0.667
Journal impact factor								
<5	2.32	1.11	Reference		<0.001	Reference		
5–10	2.18	0.95	−0.14	0.22		−0.14	0.22	0.529
10–20	2.21	1.04	−0.11	0.24		−0.23	0.25	0.355
>20	3.28	1.07	0.96	0.17		0.69	0.20	<0.001
Trial phase								
I	2.68	1.05	Reference		<0.001	Reference		
II	2.70	1.18	0.02	0.15		-0.21	0.16	0.183
III	3.36	1.22	0.68	0.17		0.20	0.18	0.275
Source of trial funding								
No industry funding	2.39	1.15	Reference		0.021	Reference		
Industry funding	2.90	1.16	0.51	0.22		0.11	0.22	0.608
Center number								
1–12	2.50	1.07	Reference		<0.001	Reference		
13–246	3.28	1.16	0.78	0.14		0.29	0.19	0.123
Unknown	2.80	1.14	0.30	0.19		0.002	0.24	0.994
Region in which trial was conducted								
Intercontinential	3.19	1.15	Reference		<0.001	Reference		
North America	2.57	1.08	−0.62	0.15		−0.10	0.20	0.627
Others	2.34	1.10	−0.85	0.18		−0.17	0.22	0.426
Unknown	2.56	1.03	−0.63	0.29		−0.29	0.33	0.388
Tumor type								
Pan cancer	2.18	1.01	Reference		0.003	Reference		
Lung	3.05	1.20	0.86	0.28		0.39	0.29	0.179
Melanoma	3.04	1.31	0.86	0.28		0.37	0.29	0.204
Urinary system	3.05	1.05	0.87	0.30		0.28	0.31	0.360
Others	2.61	1.01	0.43	0.27		0.03	0.28	0.905
Immunotherapy strategy								
Alone	2.87	1.15	Reference		0.702	Reference		
Combined with other therapy	2.81	1.21	−0.05	0.14		−0.02	0.16	0.876
Immune checkpoint blocking agent								
Anti-CTLA-4	2.62	1.28	Reference		0.003	Reference		
Anti-PD-1	2.89	1.12	0.27	0.16		0.02	0.22	0.917
Anti-PD-L1	2.69	1.16	0.07	0.21		−0.41	0.26	0.119
Multiagents	3.50	0.90	0.88	0.25		0.29	0.26	0.268

TRIOQS-T, trial reporting of immuno-oncology quality score- toxicity score; CTLA-4, cytotoxic T-lymphocyte antigen 4; PD-1, programmed cell death protein 1; PD-L1, programmed death-ligand 1.

## Discussion

Due to the lack of guidelines for reports of IO clinical trials until TRIO statement (10, 11) came out more than 2 years ago, the reporting quality for IO clinical trials was unsatisfactory. Concerns have been raised that more structured and transparent approach was important to the benefit-risk assessment in the evaluation of IO treatment as a new therapy. Therefore, the standardized reporting is essential. This is the first systematic evaluation of the reporting quality of IO clinical trials of cancer treatment in accordance to the TRIO statement.

Immune-oncology trials presented an unsatisfied reporting quality score based on the specific 11-item scoring system derived from the TRIO statement, which consist of six-item efficacy score and five-item toxicity score. The most common reported items in traditional clinical trials are also well described in IO trials, such as response evaluation and toxicity grade. This may be attributing the success to the well-established CONSORT guidance for clinical trials. However, treatment details beyond progression and toxicity onset time and duration were poorly reported, which are crucial to evaluate the efficacy and toxicity of immunotherapy for the cancer.

Pseudo-progression, as a unique phenomenon in IO treatment, is an event that denotes the appearance of new lesions (usually with shrinkage of baseline index tumor burden) or an initial increase in index lesions with subsequent index lesion response by clinical or radiographic assessment (15, 16). Thus, IO clinical trials often allow patients to continue therapy beyond objective progression and half of publications have reported the criteria of continue treatment. However, the details of treatment after progression was seriously underreported (12.8% reported). This failure may be related to the insufficient awareness of authors on the importance of continuous therapy and may limit the ability to make a comprehensive benefit assessment.

Although specific toxicity items for IO therapy, definition of “immune-related adverse events” or “adverse events of special interest” and management of IO toxicity, were relatively well described in over half of publications, the onset and duration of IO toxicity were rare reported. It is worth noting that, unlike the traditional cancer treatment, the toxicity of IO therapy can be latency occurrence and long lasting (17, 18). Therefore, reporting the onset and duration of toxicity is arguably as clinically important to assess the risk-benefit and useful to design the subsequent IO trails.

Different from that the reporting quality of clinical trials of traditional chemotherapy has been fully evaluated since the CONSORT guideline proposed, the quality of IO clinical trials reporting has not been assessed according to the TRIO statement which was specifically designed for IO trials. This divergence may be related to the insufficient awareness of differences between IO and traditional chemotherapy clinical trials and slower uptake of the TRIO statement. Continued use of CONSORT cannot fully reflect the unique characteristics of IO clinical trials (19, 20). More advanced than CONSORT, TRIO adopts toxicity reporting standards at the initially proposed. Notably, only four trails have no description of toxicity. It is also interesting that more than half of trials published in journals of IF >20 (*n* = 173, 58%).

It is worth noting that two trial reports received the highest score of 11 points (21, 22). Both reports are multicenter randomized controlled phase III clinical studies and are published in the New England Journal of Medicine in 2015. They are both immunotherapy alone studies of nivolumab and are funded by Bristol-Myers Squibb. The tumor types they studied are melanoma (CheckMate 066) and nonsmall-cell lung cancer (CheckMate 057), respectively. Although these two trial reports meet the requirement of each item according to the TRIO statement, some subcomponents are still insufficient. Neither of them mentioned the rationale for the chosen criteria used to evaluate response to therapy and for the selection of clinical diagnoses used in data collection belong to categories such as “immune-related adverse events” or “adverse events of special interest”. Although they both reported the number (proportion) of patients who are treated beyond progression and efficacy after initial progression, they did not mention treatment beyond progression duration and emergence of new toxicity. In addition, the report of CheckMate 066 did not mention time of onset of IO toxicity.

Factors associated with higher reporting scores were also investigated. The publications in journals of IF >20 had higher quality score, either for efficacy assessment or toxicity assessment, which might be related to the original requirements and review system of the journal (13). Specific cancer, such as lung cancer and urinary system cancer had higher quality score compared with the pan cancer. Most of the trials designed for a specific cancer category aimed to confirm the clinical efficacy of the IO treatment for this disease, not just similar to the exploratory purpose in the pan cancer categories. Therefore, there were more detailed reports on the efficacy and the whole trial. Simultaneously, this would possibly reduce authors’ interest for toxicity concerns, which lead to no difference in toxicity quality score between specific cancer and pan cancer. Compared with anti-CTLA-4 agent, trials involving other agents got a better quality score, especially for efficacy score. This is largely due to the fact that other agents came out later than anti-CTLA-4 agent, when IO clinical trials were relatively mature.

Although our study comprehensively assessed the reporting quality of IO clinical trials, the limitations should also be addressed. First, this study does not compare TRIO statement with the traditional CONSORT statement, which is mainly because that the purpose of this study is to evaluate all trials of IO rather than randomized control trails. Second, the quality score in our study was given equal weight to each item on the TRIO, which may weaken some important items or overemphasize some less-important items. At last, for those recommendations with several subcomponents, we only assign values to items, not to subcomponents. This may make the evaluation criteria broad, but it is friendly and practical for most trials.

In summary, our findings show that IO trials had an unsatisfied reporting quality score assessed by TRIO statement, especially in the reporting of treatment details beyond progression and toxicity onset time and duration. High IF journals have better reporting quality. Studies focused on specific cancer and studies containing anti-PD-1 or anti-PD-L1 agents have higher efficacy quality score. As the first step toward providing an overall landscape of IO trials reporting quality, we are expecting that it may shed light into future improvement of IO trial reporting for the better benefit-risk assessment of immunotherapy.

## Data Availability Statement

The original contributions presented in the study are included in the article/**Supplementary Material**. Further inquiries can be directed to the corresponding authors.

## Author Contributions

Conceptualization: CC, SH, and LZ. Methodology: CC, YZ, and XZ. Software: YW and L-nH. Formal analysis: CC, YZ, and XZ: Data curation: CC, YZ, XZ, and ZL. Writing (original draft preparation: CC, YZ, and XZ. Writing (review and editing): all authors. All authors contributed to the article and approved the submitted version.

## Conflict of Interest

The authors declare that the research was conducted in the absence of any commercial or financial relationships that could be construed as a potential conflict of interest.

## Publisher’s Note

All claims expressed in this article are solely those of the authors and do not necessarily represent those of their affiliated organizations, or those of the publisher, the editors and the reviewers. Any product that may be evaluated in this article, or claim that may be made by its manufacturer, is not guaranteed or endorsed by the publisher.
