# Supplementary material for: Unsatisfied Reporting Quality of Clinical Trials Evaluating Immune Checkpoint Inhibitor Therapy in Cancer
Source: Front Immunol. 2021 Oct 5;12:736943. doi: 10.3389/fimmu.2021.736943 (PMC8524036; doi:10.3389/fimmu.2021.736943)
Supplement: Supplementary file 1 [file DataSheet_1.docx]

Supplementary Material

# Supplementary Data

**The List of Immuno-oncology clinical trial reports included in this study**

1. Borghaei H, Langer CJ, Gadgeel S, et al: 24-Month Overall Survival from KEYNOTE-021 Cohort G: pemetrexed and Carboplatin with or without Pembrolizumab as First-Line Therapy for Advanced Nonsquamous Non-Small Cell Lung Cancer, Journal of thoracic oncology, 2018

2. Rizvi NA, Mazieres J, Planchard D, et al: Activity and safety of nivolumab, an anti-PD-1 immune checkpoint inhibitor, for patients with advanced, refractory squamous non-small-cell lung cancer (CheckMate 063): a phase 2, single-arm trial. Lancet Oncol 16:257-65, 2015

3. Nie J, Wang C, Liu Y, et al: Addition of Low-Dose Decitabine to Anti-PD-1 Antibody Camrelizumab in Relapsed/Refractory Classical Hodgkin Lymphoma, Journal of clinical oncology, 2019, pp Jco1802151‐

4. Eggermont AM, Chiarion-Sileni V, Grob JJ, et al: Adjuvant ipilimumab versus placebo after complete resection of high-risk stage III melanoma (EORTC 18071): a randomised, double-blind, phase 3 trial, The lancet. Oncology, 2015, pp 522‐530

5. Weber J, Mandala M, Del Vecchio M, et al: Adjuvant Nivolumab versus Ipilimumab in Resected Stage III or IV Melanoma, New England journal of medicine, 2017, pp 1824‐1835

6. Eggermont AMM, Blank CU, Mandala M, et al: Adjuvant Pembrolizumab versus Placebo in Resected Stage III Melanoma, New England journal of medicine, 2018, pp 1789‐1801

7. Wrangle JM, Velcheti V, Patel MR, et al: ALT-803, an IL-15 superagonist, in combination with nivolumab in patients with metastatic non-small cell lung cancer: a non-randomised, open-label, phase 1b trial. Lancet Oncol 19:694-704, 2018

8. Robert C, Ribas A, Wolchok JD, et al: Anti-programmed-death-receptor-1 treatment with pembrolizumab in ipilimumab-refractory advanced melanoma: a randomised dose-comparison cohort of a phase 1 trial, Lancet (london, england), 2014, pp 1109‐1117

9. Ma BBY, Lim WT, Goh BC, et al: Antitumor Activity of Nivolumab in Recurrent and Metastatic Nasopharyngeal Carcinoma: An International, Multicenter Study of the Mayo Clinic Phase 2 Consortium (NCI-9742). J Clin Oncol 36:1412-1418, 2018

10. Chow LQM, Haddad R, Gupta S, et al: Antitumor activity of pembrolizumab in biomarker-unselected patients with recurrent and/or metastatic head and neck squamous cell carcinoma: results from the phase Ib KEYNOTE-012 expansion cohort, Journal of clinical oncology, 2016, pp 3838‐3845

11. Ribas A, Hamid O, Daud A, et al: Association of Pembrolizumab With Tumor Response and Survival Among Patients With Advanced Melanoma. Jama 315:1600-9, 2016

12. Schmid P, Adams S, Rugo HS, et al: Atezolizumab and Nab-Paclitaxel in Advanced Triple-Negative Breast Cancer, New England journal of medicine, 2018, pp 2108‐2121

13. Balar AV, Galsky MD, Rosenberg JE, et al: Atezolizumab as first-line treatment in cisplatin-ineligible patients with locally advanced and metastatic urothelial carcinoma: a single-arm, multicentre, phase 2 trial. Lancet 389:67-76, 2017

14. Socinski MA, Jotte RM, Cappuzzo F, et al: Atezolizumab for First-Line Treatment of Metastatic Nonsquamous NSCLC, New England journal of medicine, 2018, pp 2288‐2301

15. West H, McCleod M, Hussein M, et al: Atezolizumab in combination with carboplatin plus nab-paclitaxel chemotherapy compared with chemotherapy alone as first-line treatment for metastatic non-squamous non-small-cell lung cancer (IMpower130): a multicentre, randomised, open-label, phase 3 trial, Lancet oncology, 2019, pp 924‐937

16. Hida T, Kaji R, Satouchi M, et al: Atezolizumab in Japanese Patients With Previously Treated Advanced Non-Small-Cell Lung Cancer: a Subgroup Analysis of the Phase 3 OAK Study, Clinical lung cancer, 2018, pp e405‐e415

17. Gadgeel SM, Lukas RV, Goldschmidt J, et al: Atezolizumab in patients with advanced non-small cell lung cancer and history of asymptomatic, treated brain metastases: exploratory analyses of the phase III OAK study, Lung cancer (Amsterdam, Netherlands), 2019, pp 105‐112

18. Rosenberg JE, Hoffman-Censits J, Powles T, et al: Atezolizumab in patients with locally advanced and metastatic urothelial carcinoma who have progressed following treatment with platinum-based chemotherapy: a single-arm, multicentre, phase 2 trial. Lancet 387:1909-20, 2016

19. Reck M, Mok TSK, Nishio M, et al: Atezolizumab plus bevacizumab and chemotherapy in non-small-cell lung cancer (IMpower150): key subgroup analyses of patients with EGFR mutations or baseline liver metastases in a randomised, open-label phase 3 trial, The lancet respiratory medicine, 2019, pp 387‐401

20. Rini BI, Powles T, Atkins MB, et al: Atezolizumab plus bevacizumab versus sunitinib in patients with previously untreated metastatic renal cell carcinoma (IMmotion151): a multicentre, open-label, phase 3, randomised controlled trial, Lancet (london, england), 2019, pp 2404‐2415

21. Sullivan RJ, Hamid O, Gonzalez R, et al: Atezolizumab plus cobimetinib and vemurafenib in BRAF-mutated melanoma patients, Nature medicine, 2019, pp 929‐935

22. Gandara DR, von Pawel J, Mazieres J, et al: Atezolizumab Treatment Beyond Progression in Advanced NSCLC: results From the Randomized, Phase III OAK Study, Journal of thoracic oncology, 2018

23. Powles T, Durán I, van der Heijden MS, et al: Atezolizumab versus chemotherapy in patients with platinum-treated locally advanced or metastatic urothelial carcinoma (IMvigor211): a multicentre, open-label, phase 3 randomised controlled trial, Lancet (london, england), 2018, pp 748‐757

24. Fehrenbacher L, Spira A, Ballinger M, et al: Atezolizumab versus docetaxel for patients with previously treated non-small-cell lung cancer (POPLAR): a multicentre, open-label, phase 2 randomised controlled trial, Lancet (london, england), 2016, pp 1837‐1846

25. Rittmeyer A, Barlesi F, Waterkamp D, et al: Atezolizumab versus docetaxel in patients with previously treated non-small-cell lung cancer (OAK): a phase 3, open-label, multicentre randomised controlled trial, Lancet (london, england), 2017, pp 255‐265

26. Eng C, Kim TW, Bendell J, et al: Atezolizumab with or without cobimetinib versus regorafenib in previously treated metastatic colorectal cancer (IMblaze370): a multicentre, open-label, phase 3, randomised, controlled trial, Lancet oncology, 2019, pp 849‐861

27. McDermott DF, Sosman JA, Sznol M, et al: Atezolizumab, an Anti-Programmed Death-Ligand 1 Antibody, in Metastatic Renal Cell Carcinoma: Long-Term Safety, Clinical Activity, and Immune Correlates From a Phase Ia Study. J Clin Oncol 34:833-42, 2016

28. Da Gama Duarte J, Parakh S, Andrews MC, et al: Autoantibodies May Predict Immune-Related Toxicity: Results from a Phase I Study of Intralesional Bacillus Calmette-Guerin followed by Ipilimumab in Patients with Advanced Metastatic Melanoma. Front Immunol 9:411, 2018

29. Chung HC, Arkenau HT, Lee J, et al: Avelumab (anti-PD-L1) as first-line switch-maintenance or second-line therapy in patients with advanced gastric or gastroesophageal junction cancer: phase 1b results from the JAVELIN Solid Tumor trial, Journal for immunotherapy of cancer, 2019

30. Heery CR, O'Sullivan-Coyne G, Madan RA, et al: Avelumab for metastatic or locally advanced previously treated solid tumours (JAVELIN Solid Tumor): a phase 1a, multicohort, dose-escalation trial. Lancet Oncol 18:587-598, 2017

31. Gulley JL, Rajan A, Spigel DR, et al: Avelumab for patients with previously treated metastatic or recurrent non-small-cell lung cancer (JAVELIN Solid Tumor): dose-expansion cohort of a multicentre, open-label, phase 1b trial. Lancet Oncol 18:599-610, 2017

32. Kaufman HL, Russell J, Hamid O, et al: Avelumab in patients with chemotherapy-refractory metastatic Merkel cell carcinoma: a multicentre, single-group, open-label, phase 2 trial. Lancet Oncol 17:1374-1385, 2016

33. Motzer RJ, Penkov K, Haanen J, et al: Avelumab plus Axitinib versus Sunitinib for Advanced Renal-Cell Carcinoma, New England journal of medicine, 2019, pp 1103‐1115

34. Barlesi F, Vansteenkiste J, Spigel D, et al: Avelumab versus docetaxel in patients with platinum-treated advanced non-small-cell lung cancer (JAVELIN Lung 200): an open-label, randomised, phase 3 study, The lancet. Oncology, 2018, pp 1468‐1479

35. Dirix LY, Takacs I, Jerusalem G, et al: Avelumab, an anti-PD-L1 antibody, in patients with locally advanced or metastatic breast cancer: a phase 1b JAVELIN Solid Tumor study, Breast cancer research and treatment, 2018, pp 671‐686

36. Apolo AB, Infante JR, Balmanoukian A, et al: Avelumab, an Anti-Programmed Death-Ligand 1 Antibody, In Patients With Refractory Metastatic Urothelial Carcinoma: Results From a Multicenter, Phase Ib Study. J Clin Oncol 35:2117-2124, 2017

37. Atkins MB, Plimack ER, Puzanov I, et al: Axitinib in combination with pembrolizumab in patients with advanced renal cell cancer: a non-randomised, open-label, dose-finding, and dose-expansion phase 1b trial. Lancet Oncol 19:405-415, 2018

38. Wilky BA, Trucco MM, Subhawong TK, et al: Axitinib plus pembrolizumab in patients with advanced sarcomas including alveolar soft-part sarcoma: a single-centre, single-arm, phase 2 trial, The lancet. Oncology, 2019

39. Hodi FS, Lawrence D, Lezcano C, et al: Bevacizumab plus ipilimumab in patients with metastatic melanoma. Cancer Immunol Res 2:632-42, 2014

40. Langer CJ, Gadgeel SM, Borghaei H, et al: Carboplatin and pemetrexed with or without pembrolizumab for advanced, non-squamous non-small-cell lung cancer: a randomised, phase 2 cohort of the open-label KEYNOTE-021 study, The lancet. Oncology, 2016, pp 1497‐1508

41. Gillison ML, Blumenschein G, Fayette J, et al: CheckMate 141: 1-Year Update and Subgroup Analysis of Nivolumab as First-Line Therapy in Patients with Recurrent/Metastatic Head and Neck Cancer, Oncologist, 2018, pp 1079‐1082

42. McDermott DF, Huseni MA, Atkins MB, et al: Clinical activity and molecular correlates of response to atezolizumab alone or in combination with bevacizumab versus sunitinib in renal cell carcinoma, Nature medicine, 2018, pp 749‐757

43. Lukas RV, Rodon J, Becker K, et al: Clinical activity and safety of atezolizumab in patients with recurrent glioblastoma. J Neurooncol 140:317-328, 2018

44. Alley EW, Lopez J, Santoro A, et al: Clinical safety and activity of pembrolizumab in patients with malignant pleural mesothelioma (KEYNOTE-028): preliminary results from a non-randomised, open-label, phase 1b trial. Lancet Oncol 18:623-630, 2017

45. Long GV, Atkinson V, Lo S, et al: Combination nivolumab and ipilimumab or nivolumab alone in melanoma brain metastases: a multicentre randomised phase 2 study, The lancet. Oncology, 2018, pp 672‐681

46. Ribas A, Lawrence D, Atkinson V, et al: Combined BRAF and MEK inhibition with PD-1 blockade immunotherapy in BRAF-mutant melanoma, Nature medicine, 2019, pp 936‐940

47. D'Angelo SP, Shoushtari AN, Keohan ML, et al: Combined KIT and CTLA-4 Blockade in Patients with Refractory GIST and Other Advanced Sarcomas: A Phase Ib Study of Dasatinib plus Ipilimumab. Clin Cancer Res 23:2972-2980, 2017

48. Tawbi HA, Forsyth PA, Algazi A, et al: Combined Nivolumab and Ipilimumab in Melanoma Metastatic to the Brain. 379:722-730, 2018

49. Larkin J, Chiarion-Sileni V, Gonzalez R, et al: Combined Nivolumab and Ipilimumab or Monotherapy in Untreated Melanoma, New England journal of medicine, 2015, pp 23‐34

50. Hodi FS, Chesney J, Pavlick AC, et al: Combined nivolumab and ipilimumab versus ipilimumab alone in patients with advanced melanoma: 2-year overall survival outcomes in a multicentre, randomised, controlled, phase 2 trial, The lancet. Oncology, 2016, pp 1558‐1568

51. Weide B, Martens A, Wistuba-Hamprecht K, et al: Combined treatment with ipilimumab and intratumoral interleukin-2 in pretreated patients with stage IV melanoma-safety and efficacy in a phase II study. Cancer Immunol Immunother 66:441-449, 2017

52. Levy A, Massard C, Soria JC, et al: Concurrent irradiation with the anti-programmed cell death ligand-1 immune checkpoint blocker durvalumab: Single centre subset analysis from a phase 1/2 trial. Eur J Cancer 68:156-162, 2016

53. Ascierto PA, Ferrucci PF, Fisher R, et al: Dabrafenib, trametinib and pembrolizumab or placebo in BRAF-mutant melanoma, Nature medicine, 2019, pp 941‐946

54. Armand P, Nagler A, Weller EA, et al: Disabling immune tolerance by programmed death-1 blockade with pidilizumab after autologous hematopoietic stem-cell transplantation for diffuse large B-cell lymphoma: results of an international phase II trial. J Clin Oncol 31:4199-206, 2013

55. Kang WK, Tree AC, Jones K, et al: Dose-limiting Urinary Toxicity With Pembrolizumab Combined With Weekly Hypofractionated Radiation Therapy in Bladder Cancer. Nat Med 101:1168-1171, 2018

56. Antonia SJ, Villegas A, Daniel D, et al: Durvalumab after Chemoradiotherapy in Stage III Non-Small-Cell Lung Cancer, New England journal of medicine, 2017, pp 1919‐1929

57. Garassino MC, Cho BC, Kim JH, et al: Durvalumab as third-line or later treatment for advanced non-small-cell lung cancer (ATLANTIC): an open-label, single-arm, phase 2 study. Lancet Oncol 19:521-536, 2018

58. O'Reilly EM, Oh DY, Dhani N, et al: Durvalumab with or Without Tremelimumab for Patients with Metastatic Pancreatic Ductal Adenocarcinoma: a Phase 2 Randomized Clinical Trial, JAMA oncology, 2019

59. Tarhini AA, Lee SJ, Li X, et al: E3611—A randomized phase II study of ipilimumab at 3 or 10 mg/kg alone or in combination with high-dose interferon-a2b in advanced melanoma, Clinical cancer research, 2019, pp 524‐532

60. Theelen W, Peulen HMU, Lalezari F, et al: Effect of Pembrolizumab after Stereotactic Body Radiotherapy vs Pembrolizumab Alone on Tumor Response in Patients with Advanced Non-Small Cell Lung Cancer: results of the PEMBRO-RT Phase 2 Randomized Clinical Trial, JAMA oncology, 2019

61. Calabro L, Morra A, Fonsatti E, et al: Efficacy and safety of an intensified schedule of tremelimumab for chemotherapy-resistant malignant mesothelioma: an open-label, single-arm, phase 2 study. Lancet Respir Med 3:301-9, 2015

62. Powles T, O'Donnell PH, Massard C, et al: Efficacy and Safety of Durvalumab in Locally Advanced or Metastatic Urothelial Carcinoma: Updated Results From a Phase 1/2 Open-label Study. Lancet 3:e172411, 2017

63. Efficacy and safety of first-line avelumab treatment in patients with stage IV metastatic merkel cell carcinoma a preplanned interim analysis of a clinical trial, JAMA oncology, 2018

64. O'Day SJ, Maio M, Chiarion-Sileni V, et al: Efficacy and safety of ipilimumab monotherapy in patients with pretreated advanced melanoma: a multicenter single-arm phase II study. Ann Oncol 21:1712-7, 2010

65. Hida T, Nishio M, Nogami N, et al: Efficacy and safety of nivolumab in Japanese patients with advanced or recurrent squamous non-small cell lung cancer. Cancer Sci 108:1000-1006, 2017

66. Yamazaki N, Kiyohara Y, Uhara H, et al: Efficacy and safety of nivolumab in Japanese patients with previously untreated advanced melanoma: A phase II study. Cancer Sci 108:1223-1230, 2017

67. Bang YJ, Cho JY, Kim YH, et al: Efficacy of Sequential Ipilimumab Monotherapy versus Best Supportive Care for Unresectable Locally Advanced/Metastatic Gastric or Gastroesophageal Junction Cancer, Clinical cancer research, 2017, pp 5671‐5678

68. Daver N, Garcia-Manero G, Basu S, et al: Efficacy, safety, and biomarkers of response to azacitidine and nivolumab in relapsed/ refractory acute myeloid leukemia: a nonrandomized, open-label, phase II study, Cancer discovery, 2019, pp 370‐383

69. Le DT, Lutz E, Uram JN, et al: Evaluation of ipilimumab in combination with allogeneic pancreatic tumor cells transfected with a GM-CSF gene in previously treated pancreatic cancer, Journal of immunotherapy (hagerstown, md. : 1997), 2013, pp 382‐389

70. Hamid O, Puzanov I, Dummer R, et al: Final analysis of a randomised trial comparing pembrolizumab versus investigator-choice chemotherapy for ipilimumab-refractory advanced melanoma, European journal of cancer (oxford, england : 1990), 2017, pp 37‐45

71. Horn L, Mansfield AS, Szczęsna A, et al: First-Line Atezolizumab plus Chemotherapy in Extensive-Stage Small-Cell Lung Cancer, New England journal of medicine, 2018, pp 2220‐2229

72. Carbone DP, Reck M, Paz-Ares L, et al: First-line nivolumab in stage IV or recurrent non-small-cell lung cancer, New england journal of medicine, 2017, pp 2415‐2426

73. Balar AV, Castellano D, O'Donnell PH, et al: First-line pembrolizumab in cisplatin-ineligible patients with locally advanced and unresectable or metastatic urothelial cancer (KEYNOTE-052): a multicentre, single-arm, phase 2 study, The lancet. Oncology, 2017, pp 1483‐1492

74. Gettinger S, Horn L, Jackman D, et al: Five-Year Follow-Up of Nivolumab in Previously Treated Advanced Non-Small-Cell Lung Cancer: Results From the CA209-003 Study. J Clin Oncol 36:1675-1684, 2018

75. Maio M, Grob JJ, Aamdal S, et al: Five-year survival rates for treatment-naive patients with advanced melanoma who received ipilimumab plus dacarbazine in a phase III trial, Journal of clinical oncology, 2015, pp 1191‐1196

76. Rozeman EA, Menzies AM, van Akkooi ACJ, et al: Identification of the optimal combination dosing schedule of neoadjuvant ipilimumab plus nivolumab in macroscopic stage III melanoma (OpACIN-neo): a multicentre, phase 2, randomised, controlled trial, Lancet oncology, 2019, pp 948‐960

77. Choueiri TK, Fishman MN, Escudier B, et al: Immunomodulatory Activity of Nivolumab in Metastatic Renal Cell Carcinoma. Clin Cancer Res 22:5461-5471, 2016

78. Ben-Ami E, Barysauskas CM, Solomon S, et al: Immunotherapy with single agent nivolumab for advanced leiomyosarcoma of the uterus: Results of a phase 2 study. Cancer 123:3285-3290, 2017

79. Hodi FS, O'Day SJ, McDermott DF, et al: Improved survival with ipilimumab in patients with metastatic melanoma, New England journal of medicine, 2010, pp 711‐723

80. Herrera AF, Moskowitz AJ, Bartlett NL, et al: Interim results of brentuximab vedotin in combination with nivolumab in patients with relapsed or refractory Hodgkin lymphoma. Blood 131:1183-1194, 2018

81. Ascierto PA, Del Vecchio M, Robert C, et al: Ipilimumab 10 mg/kg versus ipilimumab 3 mg/kg in patients with unresectable or metastatic melanoma: a randomised, double-blind, multicentre, phase 3 trial, The lancet. Oncology, 2017, pp 611‐622

82. Slovin SF, Higano CS, Hamid O, et al: Ipilimumab alone or in combination with radiotherapy in metastatic castration-resistant prostate cancer: results from an open-label, multicenter phase I/II study. Ann Oncol 24:1813-21, 2013

83. Di Giacomo AM, Ascierto PA, Pilla L, et al: Ipilimumab and fotemustine in patients with advanced melanoma (NIBIT-M1): an open-label, single-arm phase 2 trial. Lancet Oncol 13:879-86, 2012

84. Davids MS, Kim HT, Bachireddy P, et al: Ipilimumab for Patients with Relapse after Allogeneic Transplantation. N Engl J Med 375:143-53, 2016

85. Reck M, Bondarenko I, Luft A, et al: Ipilimumab in combination with paclitaxel and carboplatin as first-line therapy in extensive-disease-small-cell lung cancer: results from a randomized, double-blind, multicenter phase 2 trial, Annals of oncology : official journal of the european society for medical oncology, 2013, pp 75‐83

86. Lynch TJ, Bondarenko I, Luft A, et al: Ipilimumab in combination with paclitaxel and carboplatin as first-line treatment in stage IIIB/IV non-small-cell lung cancer: results from a randomized, double-blind, multicenter phase II study, Journal of clinical oncology, 2012, pp 2046‐2054

87. Margolin K, Ernstoff MS, Hamid O, et al: Ipilimumab in patients with melanoma and brain metastases: an open-label, phase 2 trial. Lancet Oncol 13:459-65, 2012

88. Wolchok JD, Neyns B, Linette G, et al: Ipilimumab monotherapy in patients with pretreated advanced melanoma: a randomised, double-blind, multicentre, phase 2, dose-ranging study, The lancet. Oncology, 2010, pp 155‐164

89. Robert C, Thomas L, Bondarenko I, et al: Ipilimumab plus dacarbazine for previously untreated metastatic melanoma, New England journal of medicine, 2011, pp 2517‐2526

90. Hodi FS, Lee S, McDermott DF, et al: Ipilimumab plus sargramostim vs ipilimumab alone for treatment of metastatic melanoma: a randomized clinical trial, Jama, 2014, pp 1744‐1753

91. Kwon ED, Drake CG, Scher HI, et al: Ipilimumab versus placebo after radiotherapy in patients with metastatic castration-resistant prostate cancer that had progressed after docetaxel chemotherapy (CA184-043): a multicentre, randomised, double-blind, phase 3 trial, The lancet. Oncology, 2014, pp 700‐712

92. Tang C, Welsh JW, de Groot P, et al: Ipilimumab with Stereotactic Ablative Radiation Therapy: phase I Results and Immunologic Correlates from Peripheral T Cells, Clinical cancer research, 2017, pp 1388‐1396

93. Nishio M: KEYNOTE-025: Phase 1b study of pembrolizumab in Japanese patients with previously treated programmed death ligand 1-positive advanced non-small-cell lung cancer. BMC Cancer 110:1012-1020, 2019

94. Long-term Clinical Outcomes and Biomarker Analyses of Atezolizumab Therapy for Patients with Metastatic Triple-Negative Breast Cancer: a Phase 1 Study, JAMA oncology, 2018

95. Yamazaki N: Long-term follow up of nivolumab in previously untreated Japanese patients with advanced or recurrent malignant melanoma. Nat Med 110:1995-2003, 2019

96. Liu SV, Camidge DR, Gettinger SN, et al: Long-term survival follow-up of atezolizumab in combination with platinum-based doublet chemotherapy in patients with advanced non-small-cell lung cancer, European journal of cancer (oxford, england : 1990), 2018, pp 114‐122

97. Ralph C, Elkord E, Burt DJ, et al: Modulation of lymphocyte regulation for cancer therapy: a phase II trial of tremelimumab in advanced gastric and esophageal adenocarcinoma. Clin Cancer Res 16:1662-72, 2010

98. Powles T, Eder JP, Fine GD, et al: MPDL3280A (anti-PD-L1) treatment leads to clinical activity in metastatic bladder cancer. Nature 515:558-62, 2014

99. Maruyama D, Hatake K, Kinoshita T, et al: Multicenter phase II study of nivolumab in Japanese patients with relapsed or refractory classical Hodgkin lymphoma. Cancer Sci 108:1007-1012, 2017

100. Amaria RN, Reddy SM, Tawbi HA, et al: Neoadjuvant immune checkpoint blockade in high-risk resectable melanoma, Nature medicine, 2018, pp 1649‐1654

101. Tarhini A, Lin Y, Lin H, et al: Neoadjuvant ipilimumab (3 mg/kg or 10 mg/kg) and high dose IFN-alpha2b in locally/regionally advanced melanoma: safety, efficacy and impact on T-cell repertoire, Journal for immunotherapy of cancer, 2018, pp 112

102. Schalper KA, Rodriguez-Ruiz ME, Diez-Valle R, et al: Neoadjuvant nivolumab modifies the tumor immune microenvironment in resectable glioblastoma. Nat Med 25:470-476, 2019

103. Blank CU, Rozeman EA, Fanchi LF, et al: Neoadjuvant versus adjuvant ipilimumab plus nivolumab in macroscopic stage III melanoma, Nature medicine, 2018, pp 1655‐1661

104. Antonia SJ, Lopez-Martin JA, Bendell J, et al: Nivolumab alone and nivolumab plus ipilimumab in recurrent small-cell lung cancer (CheckMate 032): a multicentre, open-label, phase 1/2 trial. Lancet Oncol 17:883-895, 2016

105. Postow MA, Chesney J, Pavlick AC, et al: Nivolumab and ipilimumab versus ipilimumab in untreated melanoma, New England journal of medicine, 2015, pp 2006‐2017

106. Younes A, Santoro A, Shipp M, et al: Nivolumab for classical Hodgkin's lymphoma after failure of both autologous stem-cell transplantation and brentuximab vedotin: a multicentre, multicohort, single-arm phase 2 trial. Lancet Oncol 17:1283-94, 2016

107. Motzer RJ, Rini BI, McDermott DF, et al: Nivolumab for Metastatic Renal Cell Carcinoma: results of a Randomized Phase II Trial, Journal of clinical oncology, 2015, pp 1430‐1437

108. Morris VK, Salem ME, Nimeiri H, et al: Nivolumab for previously treated unresectable metastatic anal cancer (NCI9673): a multicentre, single-arm, phase 2 study. Lancet Oncol 18:446-453, 2017

109. Ferris RL, Blumenschein G, Fayette J, et al: Nivolumab for Recurrent Squamous-Cell Carcinoma of the Head and Neck, New England journal of medicine, 2016, pp 1856‐1867

110. Armand P, Engert A, Younes A, et al: Nivolumab for Relapsed/Refractory Classic Hodgkin Lymphoma After Failure of Autologous Hematopoietic Cell Transplantation: Extended Follow-Up of the Multicohort Single-Arm Phase II CheckMate 205 Trial. J Clin Oncol 36:1428-1439, 2018

111. Lee JS, Lee KH, Cho EK, et al: Nivolumab in advanced non-small-cell lung cancer patients who failed prior platinum-based chemotherapy. Lung Cancer 122:234-242, 2018

112. Rizvi NA, Hellmann MD, Brahmer JR, et al: Nivolumab in Combination With Platinum-Based Doublet Chemotherapy for First-Line Treatment of Advanced Non-Small-Cell Lung Cancer. J Clin Oncol 34:2969-79, 2016

113. Sharma P, Retz M, Siefker-Radtke A, et al: Nivolumab in metastatic urothelial carcinoma after platinum therapy (CheckMate 275): a multicentre, single-arm, phase 2 trial. Lancet Oncol 18:312-322, 2017

114. Kang YK, Boku N, Satoh T, et al: Nivolumab in patients with advanced gastric or gastro-oesophageal junction cancer refractory to, or intolerant of, at least two previous chemotherapy regimens (ONO-4538-12, ATTRACTION-2): a randomised, double-blind, placebo-controlled, phase 3 trial, Lancet (london, england), 2017, pp 2461‐2471

115. El-Khoueiry AB, Sangro B, Yau T, et al: Nivolumab in patients with advanced hepatocellular carcinoma (CheckMate 040): an open-label, non-comparative, phase 1/2 dose escalation and expansion trial, Lancet, 2017

116. Overman MJ, McDermott R, Leach JL, et al: Nivolumab in patients with metastatic DNA mismatch repair-deficient or microsatellite instability-high colorectal cancer (CheckMate 142): an open-label, multicentre, phase 2 study, The lancet. Oncology, 2017, pp 1182‐1191

117. Ferris RL, Licitra L, Fayette J, et al: Nivolumab in Patients With Recurrent or Metastatic Squamous Cell Carcinoma of the Head and Neck: efficacy and Safety in CheckMate 141 by Prior Cetuximab Use, Clinical cancer research, 2019

118. Lesokhin AM, Ansell SM, Armand P, et al: Nivolumab in Patients With Relapsed or Refractory Hematologic Malignancy: Preliminary Results of a Phase Ib Study. J Clin Oncol 34:2698-704, 2016

119. Robert C, Long GV, Brady B, et al: Nivolumab in previously untreated melanoma without BRAF mutation, New England journal of medicine, 2015, pp 320‐330

120. Murakami H, Kenmotsu H, Fujiwara Y, et al: Nivolumab Monotherapy for First-Line Treatment of Advanced Non-Small-Cell Lung Cancer. Invest New Drugs 34:2980-7, 2016

121. Sharma P, Callahan MK, Bono P, et al: Nivolumab monotherapy in recurrent metastatic urothelial carcinoma (CheckMate 032): a multicentre, open-label, two-stage, multi-arm, phase 1/2 trial. World J Urol 17:1590-1598, 2016

122. Scherpereel A, Mazieres J, Greillier L, et al: Nivolumab or nivolumab plus ipilimumab in patients with relapsed malignant pleural mesothelioma (IFCT-1501 MAPS2): a multicentre, open-label, randomised, non-comparative, phase 2 trial, Lancet oncology, 2019, pp 239‐253

123. Gettinger S, Hellmann MD, Chow LQM, et al: Nivolumab Plus Erlotinib in Patients With EGFR-Mutant Advanced NSCLC. Mol Ther 13:1363-1372, 2018

124. Hellmann MD, Rizvi NA, Goldman JW, et al: Nivolumab plus ipilimumab as first-line treatment for advanced non-small-cell lung cancer (CheckMate 012): results of an open-label, phase 1, multicohort study, The lancet. Oncology, 2017, pp 31‐41

125. Wolchok JD, Kluger H, Callahan MK, et al: Nivolumab plus ipilimumab in advanced melanoma. N Engl J Med 369:122-33, 2013

126. Hellmann MD, Ciuleanu TE, Pluzanski A, et al: Nivolumab plus Ipilimumab in Lung Cancer with a High Tumor Mutational Burden, New England journal of medicine, 2018, pp 2093‐2104

127. Greenberg PD, Callahan MK, Kluger H, et al: Nivolumab Plus Ipilimumab in Patients With Advanced Melanoma: Updated Survival, Response, and Safety Data in a Phase I Dose-Escalation Study. Blood 36:391-398, 2018

128. Hodi FS, Chiarion-Sileni V, Gonzalez R, et al: Nivolumab plus ipilimumab or nivolumab alone versus ipilimumab alone in advanced melanoma (CheckMate 067): 4-year outcomes of a multicentre, randomised, phase 3 trial, The lancet. Oncology, 2018, pp 1480‐1492

129. Motzer RJ, Tannir NM, McDermott DF, et al: Nivolumab plus Ipilimumab versus Sunitinib in Advanced Renal-Cell Carcinoma, New England journal of medicine, 2018, pp 1277‐1290

130. Haddad R, Concha-Benavente F, Blumenschein G, et al: Nivolumab treatment beyond RECIST-defined progression in recurrent or metastatic squamous cell carcinoma of the head and neck in CheckMate 141: a subgroup analysis of a randomized phase 3 clinical trial, Cancer, 2019

131. Kudo T, Hamamoto Y, Kato K, et al: Nivolumab treatment for oesophageal squamous-cell carcinoma: an open-label, multicentre, phase 2 trial. Lancet Oncol 18:631-639, 2017

132. Weber JS, D'Angelo SP, Minor D, et al: Nivolumab versus chemotherapy in patients with advanced melanoma who progressed after anti-CTLA-4 treatment (CheckMate 037): a randomised, controlled, open-label, phase 3 trial, The lancet. Oncology, 2015, pp 375‐384

133. Borghaei H, Paz-Ares L, Horn L, et al: Nivolumab versus Docetaxel in Advanced Nonsquamous Non-Small-Cell Lung Cancer, New England journal of medicine, 2015, pp 1627‐1639

134. Brahmer J, Reckamp KL, Baas P, et al: Nivolumab versus Docetaxel in Advanced Squamous-Cell Non-Small-Cell Lung Cancer, New England journal of medicine, 2015, pp 123‐135

135. Tomita Y, Fukasawa S, Shinohara N, et al: Nivolumab versus everolimus in advanced renal cell carcinoma: japanese subgroup 3-year follow-up analysis from the Phase III CheckMate 025 study, Japanese journal of clinical oncology, 2019, pp 506‐514

136. Tomita Y, Fukasawa S, Shinohara N, et al: Nivolumab versus everolimus in advanced renal cell carcinoma: japanese subgroup analysis from the CheckMate 025 study, Japanese journal of clinical oncology, 2017, pp 639‐646

137. Motzer RJ, Escudier B, McDermott DF, et al: Nivolumab versus Everolimus in Advanced Renal-Cell Carcinoma, New England journal of medicine, 2015, pp 1803‐1813

138. Ferris RL, Blumenschein G, Fayette J, et al: Nivolumab vs investigator's choice in recurrent or metastatic squamous cell carcinoma of the head and neck: 2-year long-term survival update of CheckMate 141 with analyses by tumor PD-L1 expression, Oral oncology, 2018, pp 45‐51

139. Omuro A, Vlahovic G, Lim M, et al: Nivolumab with or without ipilimumab in patients with recurrent glioblastoma: results from exploratory phase I cohorts of CheckMate 143, Neuro-oncology, 2018, pp 674‐686

140. D'Angelo SP, Mahoney MR, Van Tine BA, et al: Nivolumab with or without ipilimumab treatment for metastatic sarcoma (Alliance A091401): two open-label, non-comparative, randomised, phase 2 trials, The lancet. Oncology, 2018, pp 416‐426

141. Zimmer L, Eigentler TK, Kiecker F, et al: Open-label, multicenter, single-arm phase II DeCOG-study of ipilimumab in pretreated patients with different subtypes of metastatic melanoma. J Transl Med 13:351, 2015

142. Chih-Hsin Yang J, Shepherd FA, Kim DW, et al: Osimertinib Plus Durvalumab versus Osimertinib Monotherapy in EGFR T790M–Positive NSCLC following Previous EGFR TKI Therapy: CAURAL Brief Report, Journal of thoracic oncology, 2019

143. Arriola E, Wheater M, Galea I, et al: Outcome and Biomarker Analysis from a Multicenter Phase 2 Study of Ipilimumab in Combination with Carboplatin and Etoposide as First-Line Therapy for Extensive-Stage SCLC. J Thorac Oncol 11:1511-21, 2016

144. Carlino MS, Long GV, Schadendorf D, et al: Outcomes by line of therapy and programmed death ligand 1 expression in patients with advanced melanoma treated with pembrolizumab or ipilimumab in KEYNOTE-006: a randomised clinical trial, European journal of cancer (Oxford, England : 1990), 2018, pp 236‐243

145. Gettinger SN, Horn L, Gandhi L, et al: Overall survival and long-term safety of nivolumab (anti-programmed death 1 antibody, BMS-936558, ONO-4538) in patients with previously treated advanced non-small-cell lung cancer, Journal of clinical oncology, 2015, pp 2004‐2012

146. Larkin J, Minor D, D'Angelo S, et al: Overall Survival in Patients With Advanced Melanoma Who Received Nivolumab Versus Investigator's Choice Chemotherapy in CheckMate 037: a Randomized, Controlled, Open-Label Phase III Trial, Journal of clinical oncology, 2018, pp 383‐390

147. Wolchok JD, Chiarion-Sileni V, Gonzalez R, et al: Overall Survival with Combined Nivolumab and Ipilimumab in Advanced Melanoma, New England journal of medicine, 2017, pp 1345‐1356

148. Antonia SJ, Villegas A, Daniel D, et al: Overall Survival with Durvalumab after Chemoradiotherapy in Stage III NSCLC, New England journal of medicine, 2018, pp 2342‐2350

149. Le DT, Uram JN, Wang H, et al: PD-1 Blockade in Tumors with Mismatch-Repair Deficiency. N Engl J Med 372:2509-20, 2015

150. Ansell SM, Lesokhin AM, Borrello I, et al: PD-1 blockade with nivolumab in relapsed or refractory Hodgkin's lymphoma. N Engl J Med 372:311-9, 2015

151. Armand P, Chen YB, Redd RA, et al: PD-1 Blockade with Pembrolizumab for Classical Hodgkin Lymphoma after Autologous Stem Cell Transplantation, Blood, 2019

152. Nghiem PT, Bhatia S, Lipson EJ, et al: PD-1 Blockade with Pembrolizumab in Advanced Merkel-Cell Carcinoma. N Engl J Med 374:2542-52, 2016

153. Gadgeel SM, Stevenson JP, Langer CJ, et al: Pembrolizumab and platinum-based chemotherapy as first-line therapy for advanced non-small-cell lung cancer: Phase 1 cohorts from the KEYNOTE-021 study. Lung Cancer 125:273-281, 2018

154. Hui R, Garon EB, Goldman JW, et al: Pembrolizumab as first-line therapy for patients with PD-L1-positive advanced non-small cell lung cancer: a phase 1 trial, Annals of oncology : official journal of the european society for medical oncology, 2017, pp 874‐881

155. Bellmunt J, de Wit R, Vaughn DJ, et al: Pembrolizumab as Second-Line Therapy for Advanced Urothelial Carcinoma, New England journal of medicine, 2017, pp 1015‐1026

156. Parikh M, Pan CX, Beckett LA, et al: Pembrolizumab Combined With Either Docetaxel or Gemcitabine in Patients With Advanced or Metastatic Platinum-Refractory Urothelial Cancer: results From a Phase I Study, Clinical genitourinary cancer, 2018, pp 421‐428.e1

157. Goldberg SB, Gettinger SN, Mahajan A, et al: Pembrolizumab for patients with melanoma or non-small-cell lung cancer and untreated brain metastases: early analysis of a non-randomised, open-label, phase 2 trial. Lancet Oncol 17:976-983, 2016

158. Muro K, Chung HC, Shankaran V, et al: Pembrolizumab for patients with PD-L1-positive advanced gastric cancer (KEYNOTE-012): a multicentre, open-label, phase 1b trial. Blood 17:717-726, 2016

159. Rodriguez MA, Cabanillas F, Fowler NH, et al: Pembrolizumab for Platinum- and Cetuximab-Refractory Head and Neck Cancer: Results From a Single-Arm, Phase II Study. Br J Haematol 35:1542-1549, 2017

160. Garon EB, Rizvi NA, Hui R, et al: Pembrolizumab for the treatment of non-small-cell lung cancer. N Engl J Med 372:2018-28, 2015

161. Tawbi HA, Burgess M, Bolejack V, et al: Pembrolizumab in advanced soft-tissue sarcoma and bone sarcoma (SARC028): a multicentre, two-cohort, single-arm, open-label, phase 2 trial, The lancet. Oncology, 2017, pp 1493‐1501

162. Tahara M: Pembrolizumab in Asia-Pacific patients with advanced head and neck squamous cell carcinoma: Analyses from KEYNOTE-012. Pediatr Blood Cancer 109:771-776, 2018

163. Zhu AX, Finn RS, Edeline J, et al: Pembrolizumab in patients with advanced hepatocellular carcinoma previously treated with sorafenib (KEYNOTE-224): a non-randomised, open-label phase 2 trial. Lancet Oncol 19:940-952, 2018

164. Leighl NB, Hellmann MD, Hui R, et al: Pembrolizumab in patients with advanced non-small-cell lung cancer (KEYNOTE-001): 3-year results from an open-label, phase 1 study, The lancet respiratory medicine, 2019, pp 347‐357

165. Nanda R, Chow LQ, Dees EC, et al: Pembrolizumab in Patients With Advanced Triple-Negative Breast Cancer: Phase Ib KEYNOTE-012 Study. Lancet Oncol 34:2460-7, 2016

166. Ding W, LaPlant BR, Call TG, et al: Pembrolizumab in patients with CLL and Richter transformation or with relapsed CLL. J Transl Med 129:3419-3427, 2017

167. Ott PA, Elez E, Hiret S, et al: Pembrolizumab in Patients With Extensive-Stage Small-Cell Lung Cancer: Results From the Phase Ib KEYNOTE-028 Study. J Clin Oncol 35:3823-3829, 2017

168. Voronov E, Apte RN, Varga A, et al: Pembrolizumab in patients with programmed death ligand 1-positive advanced ovarian cancer: Analysis of KEYNOTE-028. Proc Natl Acad Sci U S A 152:243-250, 2019

169. Giaccone G, Kim C, Thompson J, et al: Pembrolizumab in patients with thymic carcinoma: a single-arm, single-centre, phase 2 study. Lancet Oncol 19:347-355, 2018

170. Rini BI, Plimack ER, Stus V, et al: Pembrolizumab plus Axitinib versus Sunitinib for Advanced Renal-Cell Carcinoma, New England journal of medicine, 2019, pp 1116‐1127

171. Paz-Ares L, Luft A, Vicente D, et al: Pembrolizumab plus Chemotherapy for Squamous Non-Small-Cell Lung Cancer, New England journal of medicine, 2018, pp 2040‐2051

172. Gandhi L, Rodríguez-Abreu D, Gadgeel S, et al: Pembrolizumab plus Chemotherapy in Metastatic Non-Small-Cell Lung Cancer, New England journal of medicine, 2018, pp 2078‐2092

173. Reck M, Rodríguez-Abreu D, Robinson AG, et al: Pembrolizumab versus Chemotherapy for PD-L1-Positive Non-Small-Cell Lung Cancer, New England journal of medicine, 2016, pp 1823‐1833

174. Mok TSK, Wu YL, Kudaba I, et al: Pembrolizumab versus chemotherapy for previously untreated, PD-L1-expressing, locally advanced or metastatic non-small-cell lung cancer (KEYNOTE-042): a randomised, open-label, controlled, phase 3 trial, Lancet (london, england), 2019, pp 1819‐1830

175. Herbst RS, Baas P, Kim DW, et al: Pembrolizumab versus docetaxel for previously treated, PD-L1-positive, advanced non-small-cell lung cancer (KEYNOTE-010): a randomised controlled trial, Lancet (london, england), 2016, pp 1540‐1550

176. Ribas A, Puzanov I, Dummer R, et al: Pembrolizumab versus investigator-choice chemotherapy for ipilimumab-refractory melanoma (KEYNOTE-002): a randomised, controlled, phase 2 trial, The lancet. Oncology, 2015, pp 908‐918

177. Schachter J, Ribas A, Long GV, et al: Pembrolizumab versus ipilimumab for advanced melanoma: final overall survival results of a multicentre, randomised, open-label phase 3 study (KEYNOTE-006), Lancet (london, england), 2017, pp 1853‐1862

178. Robert C, Schachter J, Long GV, et al: Pembrolizumab versus Ipilimumab in Advanced Melanoma, New England journal of medicine, 2015, pp 2521‐2532

179. Cohen EEW, Soulières D, Le Tourneau C, et al: Pembrolizumab versus methotrexate, docetaxel, or cetuximab for recurrent or metastatic head-and-neck squamous cell carcinoma (KEYNOTE-040): a randomised, open-label, phase 3 study, Lancet (london, england), 2019, pp 156‐167

180. Shitara K, Özgüroğlu M, Bang YJ, et al: Pembrolizumab versus paclitaxel for previously treated, advanced gastric or gastro-oesophageal junction cancer (KEYNOTE-061): a randomised, open-label, controlled, phase 3 trial, Lancet (london, england), 2018, pp 123‐133

181. Sundahl N, De Wolf K, Kruse V, et al: Phase 1 Dose Escalation Trial of Ipilimumab and Stereotactic Body Radiation Therapy in Metastatic Melanoma. Int J Radiat Oncol Biol Phys 100:906-915, 2018

182. Rini BI, Stein M, Shannon P, et al: Phase 1 dose-escalation trial of tremelimumab plus sunitinib in patients with metastatic renal cell carcinoma. Cancer 117:758-67, 2011

183. Williams NL, Wuthrick EJ, Kim H, et al: Phase 1 Study of Ipilimumab Combined With Whole Brain Radiation Therapy or Radiosurgery for Melanoma Patients With Brain Metastases. Int J Radiat Oncol Biol Phys 99:22-30, 2017

184. Shimizu T, Seto T, Hirai F, et al: Phase 1 study of pembrolizumab (MK-3475; anti-PD-1 monoclonal antibody) in Japanese patients with advanced solid tumors. Invest New Drugs 34:347-54, 2016

185. Gibney GT, Hamid O, Lutzky J, et al: Phase 1/2 study of epacadostat in combination with ipilimumab in patients with unresectable or metastatic melanoma, Journal for immunotherapy of cancer, 2019

186. Spigel DR, Reynolds C, Waterhouse D, et al: Phase 1/2 Study of the Safety and Tolerability of Nivolumab Plus Crizotinib for the First-Line Treatment of Anaplastic Lymphoma Kinase Translocation - Positive Advanced Non-Small Cell Lung Cancer (CheckMate 370). J Thorac Oncol 13:682-688, 2018

187. Yamazaki N, Takenouchi T, Fujimoto M, et al: Phase 1b study of pembrolizumab (MK-3475; anti-PD-1 monoclonal antibody) in Japanese patients with advanced melanoma (KEYNOTE-041). Cancer Chemother Pharmacol 79:651-660, 2017

188. Hong D, Rasco D, Veeder M, et al: A Phase 1b/2 Study of the Bruton Tyrosine Kinase Inhibitor Ibrutinib and the PD-L1 Inhibitor Durvalumab in Patients with Pretreated Solid Tumors. Oncology 97:102-111, 2019

189. Joshua AM, Monzon JG, Mihalcioiu C, et al: A phase 2 study of tremelimumab in patients with advanced uveal melanoma. Melanoma Res 25:342-7, 2015

190. Galsky MD, Wang H, Hahn NM, et al: Phase 2 Trial of Gemcitabine, Cisplatin, plus Ipilimumab in Patients with Metastatic Urothelial Cancer and Impact of DNA Damage Response Gene Mutations on Outcomes. J Immunother Cancer 73:751-759, 2018

191. Royal RE, Levy C, Turner K, et al: Phase 2 trial of single agent Ipilimumab (anti-CTLA-4) for locally advanced or metastatic pancreatic adenocarcinoma. J Immunother 33:828-33, 2010

192. Reilley MJ, Bailey A, Subbiah V, et al: Phase I clinical trial of combination imatinib and ipilimumab in patients with advanced malignancies. J Immunother Cancer 5:35, 2017

193. Merchant MS, Wright M, Baird K, et al: Phase I Clinical Trial of Ipilimumab in Pediatric Patients with Advanced Solid Tumors. Clin Cancer Res 22:1364-70, 2016

194. Aglietta M, Barone C, Sawyer MB, et al: A phase I dose escalation trial of tremelimumab (CP-675,206) in combination with gemcitabine in chemotherapy-naive patients with metastatic pancreatic cancer. Ann Oncol 25:1750-5, 2014

195. Sakamuri D, Glitza IC, Betancourt Cuellar SL, et al: Phase I Dose-Escalation Study of Anti-CTLA-4 Antibody Ipilimumab and Lenalidomide in Patients with Advanced Cancers. Mol Cancer Ther 17:671-676, 2018

196. Mizugaki H, Yamamoto N: Phase I dose-finding study of monotherapy with atezolizumab, an engineered immunoglobulin monoclonal antibody targeting PD-L1, in Japanese patients with advanced solid tumors. 34:596-603, 2016

197. Ray A, Williams MA, Meek SM, et al: A phase I study of intratumoral ipilimumab and interleukin-2 in patients with advanced melanoma. Oncotarget 7:64390-64399, 2016

198. Horinouchi H, Yamamoto N, Fujiwara Y, et al: Phase I study of ipilimumab in phased combination with paclitaxel and carboplatin in Japanese patients with non-small-cell lung cancer. Invest New Drugs 33:881-9, 2015

199. Ansell SM, Hurvitz SA, Koenig PA, et al: Phase I study of ipilimumab, an anti-CTLA-4 monoclonal antibody, in patients with relapsed and refractory B-cell non-Hodgkin lymphoma, Clinical cancer research, 2009, pp 6446‐6453

200. Yamamoto N, Nokihara H, Yamada Y, et al: Phase I study of Nivolumab, an anti-PD-1 antibody, in patients with malignant solid tumors. Invest New Drugs 35:207-216, 2017

201. Patnaik A, Kang SP, Rasco D, et al: Phase I Study of Pembrolizumab (MK-3475; Anti-PD-1 Monoclonal Antibody) in Patients with Advanced Solid Tumors. Clin Cancer Res 21:4286-93, 2015

202. Brahmer JR, Drake CG, Wollner I, et al: Phase I study of single-agent anti-programmed death-1 (MDX-1106) in refractory solid tumors: safety, clinical activity, pharmacodynamics, and immunologic correlates. J Clin Oncol 28:3167-75, 2010

203. Millward M, Underhill C, Lobb S, et al: Phase I study of tremelimumab (CP-675 206) plus PF-3512676 (CPG 7909) in patients with melanoma or advanced solid tumours. Br J Cancer 108:1998-2004, 2013

204. McNeel DG, Smith HA, Eickhoff JC, et al: Phase I trial of tremelimumab in combination with short-term androgen deprivation in patients with PSA-recurrent prostate cancer. Cancer Immunol Immunother 61:1137-47, 2012

205. Weber J, Gibney G, Kudchadkar R, et al: Phase I/II Study of Metastatic Melanoma Patients Treated with Nivolumab Who Had Progressed after Ipilimumab. Cancer Immunol Res 4:345-53, 2016

206. Brohl AS, Khushalani NI, Eroglu Z, et al: A phase ib study of ipilimumab with peginterferon alfa-2b in patients with unresectable melanoma, Journal for immunotherapy of cancer, 2016

207. Weiss GJ, Waypa J, Blaydorn L, et al: A phase Ib study of pembrolizumab plus chemotherapy in patients with advanced cancer (PembroPlus). Br J Cancer 117:33-40, 2017

208. Tolcher AW, Sznol M, Hu-Lieskovan S, et al: Phase Ib Study of Utomilumab (PF-05082566), a 4-1BB/CD137 Agonist, in Combination with Pembrolizumab (MK-3475) in Patients with Advanced Solid Tumors. J Immunother Cancer 23:5349-5357, 2017

209. Weiss GJ, Blaydorn L, Beck J, et al: Phase Ib/II study of gemcitabine, nab-paclitaxel, and pembrolizumab in metastatic pancreatic adenocarcinoma. Invest New Drugs 36:96-102, 2018

210. Zimmer L, Vaubel J, Mohr P, et al: Phase II DeCOG-study of ipilimumab in pretreated and treatment-naive patients with metastatic uveal melanoma. PLoS One 10:e0118564, 2015

211. Geoerger B, Bergeron C, Gore L, et al: Phase II study of ipilimumab in adolescents with unresectable stage III or IV malignant melanoma. Eur J Cancer 86:358-363, 2017

212. Yamazaki N, Kiyohara Y, Uhara H, et al: Phase II study of ipilimumab monotherapy in Japanese patients with advanced melanoma. Cancer Chemother Pharmacol 76:997-1004, 2015

213. Patel SP, Kim DW, Bassett RL, et al: A phase II study of ipilimumab plus temozolomide in patients with metastatic melanoma. PLoS One 66:1359-1366, 2017

214. Chung KY, Gore I, Fong L, et al: Phase II study of the anti-cytotoxic T-lymphocyte-associated antigen 4 monoclonal antibody, tremelimumab, in patients with refractory metastatic colorectal cancer. J Clin Oncol 28:3485-90, 2010

215. Chen R, Zinzani PL, Fanale MA, et al: Phase II Study of the Efficacy and Safety of Pembrolizumab for Relapsed/Refractory Classic Hodgkin Lymphoma. J Clin Oncol 35:2125-2132, 2017

216. Yamazaki N, Uhara H, Fukushima S, et al: Phase II study of the immune-checkpoint inhibitor ipilimumab plus dacarbazine in Japanese patients with previously untreated, unresectable or metastatic melanoma. Cancer Chemother Pharmacol 76:969-75, 2015

217. Amin A, Lawson DH, Salama AKS, et al: Phase II study of vemurafenib followed by ipilimumab in patients with previously untreated BRAF-mutated metastatic melanoma, Journal for immunotherapy of cancer, 2016

218. Peters S, Gettinger S, Johnson ML, et al: Phase II Trial of Atezolizumab As First-Line or Subsequent Therapy for Patients With Programmed Death-Ligand 1-Selected Advanced Non-Small-Cell Lung Cancer (BIRCH). J Clin Oncol 35:2781-2789, 2017

219. Haag GM, Zoernig I, Hassel JC, et al: Phase II trial of ipilimumab in melanoma patients with preexisting humoural immune response to NY-ESO-1. Biomed Res Int 90:122-129, 2018

220. Adra N, Einhorn LH, Althouse SK, et al: Phase II trial of pembrolizumab in patients with platinum refractory germ-cell tumors: a Hoosier Cancer Research Network Study GU14-206. Ann Oncol 29:209-214, 2018

221. Kirkwood JM, Lorigan P, Hersey P, et al: Phase II trial of tremelimumab (CP-675,206) in patients with advanced refractory or relapsed melanoma. Clin Cancer Res 16:1042-8, 2010

222. Ribas A, Kefford R, Marshall MA, et al: Phase III randomized clinical trial comparing tremelimumab with standard-of-care chemotherapy in patients with advanced melanoma, Journal of clinical oncology, 2013, pp 616‐622

223. Reck M, Luft A, Szczesna A, et al: Phase III Randomized Trial of Ipilimumab Plus Etoposide and Platinum Versus Placebo Plus Etoposide and Platinum in Extensive-Stage Small-Cell Lung Cancer, Journal of clinical oncology, 2016, pp 3740‐3748

224. Govindan R, Szczesna A, Ahn MJ, et al: Phase III trial of ipilimumab combined with paclitaxel and carboplatin in advanced squamous non–small-cell lung cancer, Journal of clinical oncology, 2017, pp 3449‐3457

225. Bang YJ, Ruiz EY, Van Cutsem E, et al: Phase III, randomised trial of avelumab versus physician's choice of chemotherapy as third-line treatment of patients with advanced gastric or gastro-oesophageal junction cancer: primary analysis of JAVELIN Gastric 300, Annals of oncology : official journal of the european society for medical oncology, 2018, pp 2052‐2060

226. Herbst RS, Soria JC, Kowanetz M, et al: Predictive correlates of response to the anti-PD-L1 antibody MPDL3280A in cancer patients. Nature 515:563-7, 2014

227. Choueiri TK, Larkin J, Oya M, et al: Preliminary results for avelumab plus axitinib as first-line therapy in patients with advanced clear-cell renal-cell carcinoma (JAVELIN Renal 100): an open-label, dose-finding and dose-expansion, phase 1b trial. Lancet Oncol 19:451-460, 2018

228. Carthon BC, Wolchok JD, Yuan J, et al: Preoperative CTLA-4 blockade: tolerability and immune monitoring in the setting of a presurgical clinical trial. Clin Cancer Res 16:2861-71, 2010

229. Shaverdian N, Lisberg AE, Bornazyan K, et al: Previous radiotherapy and the clinical activity and toxicity of pembrolizumab in the treatment of non-small-cell lung cancer: a secondary analysis of the KEYNOTE-001 phase 1 trial, Lancet oncology, 2017, pp 895‐903

230. Armand P, Shipp MA, Ribrag V, et al: Programmed Death-1 Blockade With Pembrolizumab in Patients With Classical Hodgkin Lymphoma After Brentuximab Vedotin Failure. J Clin Oncol 34:3733-3739, 2016

231. Quispel-Janssen J, van der Noort V, de Vries JF, et al: Programmed Death 1 Blockade With Nivolumab in Patients With Recurrent Malignant Pleural Mesothelioma. J Thorac Oncol 13:1569-1576, 2018

232. Daud AI, Wolchok JD, Robert C, et al: Programmed Death-Ligand 1 Expression and Response to the Anti-Programmed Death 1 Antibody Pembrolizumab in Melanoma. J Clin Oncol 34:4102-4109, 2016

233. Eggermont AM, Chiarion-Sileni V, Grob JJ, et al: Prolonged Survival in Stage III Melanoma with Ipilimumab Adjuvant Therapy, New England journal of medicine, 2016, pp 1845‐1855

234. Hiniker SM, Reddy SA, Maecker HT, et al: A Prospective Clinical Trial Combining Radiation Therapy With Systemic Immunotherapy in Metastatic Melanoma, International journal of radiation oncology, biology, physics, 2016, pp 578‐588

235. Hamid O, Schmidt H, Nissan A, et al: A prospective phase II trial exploring the association between tumor microenvironment biomarkers and clinical activity of ipilimumab in advanced melanoma, Journal of translational medicine, 2011, pp 204

236. Levy BP, Giaccone G, Besse B, et al: Randomised phase 2 study of pembrolizumab plus CC-486 versus pembrolizumab plus placebo in patients with previously treated advanced non-small cell lung cancer, European journal of cancer, 2019, pp 120‐128

237. Loibl S, Untch M, Burchardi N, et al: A randomised phase II study investigating durvalumab in addition to an anthracycline taxane-based neoadjuvant therapy in early triple negative breast cancer - clinical results and biomarker analysis of GeparNuevo study, Annals of oncology : official journal of the european society for medical oncology, 2019

238. Pujol JL, Greillier L, Audigier-Valette C, et al: A Randomized Non-Comparative Phase II Study of Anti-Programmed Cell Death-Ligand 1 Atezolizumab or Chemotherapy as Second-Line Therapy in Patients With Small Cell Lung Cancer: results From the IFCT-1603 Trial, Journal of thoracic oncology, 2019, pp 903‐913

239. Weber J, Hamid O, Amin A, et al: Randomized phase I pharmacokinetic study of ipilimumab with or without one of two different chemotherapy regimens in patients with untreated advanced melanoma, Cancer immunity, 2013, pp 7

240. Fradet Y, Bellmunt J, Vaughn DJ, et al: Randomized phase III KEYNOTE-045 trial of pembrolizumab versus paclitaxel, docetaxel, or vinflunine in recurrent advanced urothelial cancer: results of > 2 years of follow-up, Annals of oncology : official journal of the european society for medical oncology, 2019

241. Beer TM, Kwon ED, Drake CG, et al: Randomized, Double-Blind, Phase III Trial of Ipilimumab Versus Placebo in Asymptomatic or Minimally Symptomatic Patients With Metastatic Chemotherapy-Naive Castration-Resistant Prostate Cancer, Journal of clinical oncology, 2017, pp 40‐47

242. Weber J, Thompson JA, Hamid O, et al: A randomized, double-blind, placebo-controlled, phase II study comparing the tolerability and efficacy of ipilimumab administered with or without prophylactic budesonide in patients with unresectable stage III or IV melanoma, Clinical cancer research, 2009, pp 5591‐5598

243. Chesney J, Puzanov I, Collichio F, et al: Randomized, Open-Label Phase II Study Evaluating the Efficacy and Safety of Talimogene Laherparepvec in Combination With Ipilimumab Versus Ipilimumab Alone in Patients With Advanced, Unresectable Melanoma, Journal of clinical oncology, 2018, pp 1658‐1667

244. Kiyota N, Hasegawa Y, Takahashi S, et al: A randomized, open-label, Phase III clinical trial of nivolumab vs. therapy of investigator's choice in recurrent squamous cell carcinoma of the head and neck: a subanalysis of Asian patients versus the global population in checkmate 141, Oral oncology, 2017, pp 138‐146

245. Ariyan CE, Brady MS, Siegelbaum RH, et al: Robust Antitumor Responses Result from Local Chemotherapy and CTLA-4 Blockade. Pediatr Blood Cancer 6:189-200, 2018

246. Brahmer JR, Tykodi SS, Chow LQ, et al: Safety and activity of anti-PD-L1 antibody in patients with advanced cancer. N Engl J Med 366:2455-65, 2012

247. Younes A, Brody J, Carpio C, et al: Safety and activity of ibrutinib in combination with nivolumab in patients with relapsed non-Hodgkin lymphoma or chronic lymphocytic leukaemia: a phase 1/2a study. Lancet Haematol 6:e67-e78, 2019

248. Plimack ER, Bellmunt J, Gupta S, et al: Safety and activity of pembrolizumab in patients with locally advanced or metastatic urothelial cancer (KEYNOTE-012): a non-randomised, open-label, phase 1b study. Lancet Oncol 18:212-220, 2017

249. Hamanishi J, Mandai M, Ikeda T, et al: Safety and Antitumor Activity of Anti-PD-1 Antibody, Nivolumab, in Patients With Platinum-Resistant Ovarian Cancer. J Clin Oncol 33:4015-22, 2015

250. Ott PA, Bang YJ, Berton-Rigaud D, et al: Safety and Antitumor Activity of Pembrolizumab in Advanced Programmed Death Ligand 1-Positive Endometrial Cancer: Results From the KEYNOTE-028 Study. J Clin Oncol 35:2535-2541, 2017

251. Hsu C, Lee SH, Ejadi S, et al: Safety and Antitumor Activity of Pembrolizumab in Patients With Programmed Death-Ligand 1-Positive Nasopharyngeal Carcinoma: Results of the KEYNOTE-028 Study. J Clin Oncol 35:4050-4056, 2017

252. Muro K, Hasegawa Y, Chung HC, et al: Safety and antitumor activity of the anti-PD-1 antibody pembrolizumab in patients with advanced colorectal carcinoma. Cancer Sci 12:e0189848, 2017

253. Mehnert JM, Varga A, Brose MS, et al: Safety and antitumor activity of the anti-PD-1 antibody pembrolizumab in patients with advanced, PD-L1-positive papillary or follicular thyroid cancer. Cancer Immunol Immunother 19:196, 2019

254. Ott PA, Piha-Paul SA, Munster P, et al: Safety and antitumor activity of the anti-PD-1 antibody pembrolizumab in patients with recurrent carcinoma of the anal canal. Ann Oncol 28:1036-1041, 2017

255. Doi T, Piha-Paul SA, Jalal SI, et al: Safety and Antitumor Activity of the Anti-Programmed Death-1 Antibody Pembrolizumab in Patients With Advanced Esophageal Carcinoma. J Clin Oncol 36:61-67, 2018

256. Antonia S, Goldberg SB, Balmanoukian A, et al: Safety and antitumour activity of durvalumab plus tremelimumab in non-small cell lung cancer: a multicentre, phase 1b study, The lancet. Oncology, 2016, pp 299‐308

257. Horn L, Gettinger SN, Gordon MS, et al: Safety and clinical activity of atezolizumab monotherapy in metastatic non-small-cell lung cancer: final results from a phase I study. Eur J Cancer 101:201-209, 2018

258. Luke JJ, Lemons JM, Karrison TG, et al: Safety and Clinical Activity of Pembrolizumab and Multisite Stereotactic Body Radiotherapy in Patients With Advanced Solid Tumors. J Clin Oncol 36:1611-1618, 2018

259. Seiwert TY, Burtness B, Mehra R, et al: Safety and clinical activity of pembrolizumab for treatment of recurrent or metastatic squamous cell carcinoma of the head and neck (KEYNOTE-012): an open-label, multicentre, phase 1b trial, The lancet. Oncology, 2016, pp 956‐965

260. Lee JM, Cimino-Mathews A, Peer CJ, et al: Safety and Clinical Activity of the Programmed Death-Ligand 1 Inhibitor Durvalumab in Combination With Poly (ADP-Ribose) Polymerase Inhibitor Olaparib or Vascular Endothelial Growth Factor Receptor 1-3 Inhibitor Cediranib in Women's Cancers: A Dose-Escalation, Phase I Study. J Clin Oncol 35:2193-2202, 2017

261. Tarhini AA, Cherian J, Moschos SJ, et al: Safety and efficacy of combination immunotherapy with interferon alfa-2b and tremelimumab in patients with stage IV melanoma. J Clin Oncol 30:322-8, 2012

262. Massard C, Gordon MS, Sharma S, et al: Safety and Efficacy of Durvalumab (MEDI4736), an Anti-Programmed Cell Death Ligand-1 Immune Checkpoint Inhibitor, in Patients With Advanced Urothelial Bladder Cancer. J Clin Oncol 34:3119-25, 2016

263. Siu LL, Even C, Mesia R, et al: Safety and Efficacy of Durvalumab with or Without Tremelimumab in Patients with PD-L1-Low/Negative Recurrent or Metastatic HNSCC: the Phase 2 CONDOR Randomized Clinical Trial, JAMA oncology, 2018

264. Kanda S, Goto K, Shiraishi H, et al: Safety and efficacy of nivolumab and standard chemotherapy drug combination in patients with advanced non-small-cell lung cancer: a four arms phase Ib study, Annals of oncology : official journal of the european society for medical oncology, 2016, pp 2242‐2250

265. Hammers HJ, Plimack ER, Infante JR, et al: Safety and Efficacy of Nivolumab in Combination With Ipilimumab in Metastatic Renal Cell Carcinoma: The CheckMate 016 Study. J Clin Oncol 35:3851-3858, 2017

266. Boku N, Ryu MH, Kato K, et al: Safety and efficacy of nivolumab in combination with S-1/capecitabine plus oxaliplatin in patients with previously untreated, unresectable, advanced, or recurrent gastric/gastroesophageal junction cancer: interim results of a randomized, phase II trial (ATTRACTION-4), Annals of oncology : official journal of the european society for medical oncology, 2019, pp 250‐258

267. George S, Motzer RJ, Hammers HJ, et al: Safety and Efficacy of Nivolumab in Patients With Metastatic Renal Cell Carcinoma Treated Beyond Progression: a Subgroup Analysis of a Randomized Clinical Trial, JAMA oncology, 2016, pp 1179‐1186

268. Frenel JS, Le Tourneau C, O'Neil B, et al: Safety and Efficacy of Pembrolizumab in Advanced, Programmed Death Ligand 1-Positive Cervical Cancer: Results From the Phase Ib KEYNOTE-028 Trial. J Clin Oncol 35:4035-4041, 2017

269. Fuchs CS, Doi T, Jang RW, et al: Safety and efficacy of pembrolizumab monotherapy in patients with previously treated advanced gastric and gastroesophageal junction cancer: phase 2 clinical KEYNOTE-059 trial, JAMA oncology, 2018

270. Gallego Perez-Larraya J, Espinos J, Idoate MA, et al: Safety and tolerability of pembrolizumab in patients with relapsed/refractory primary mediastinal large B-cell lymphoma. J Transl Med 130:267-270, 2017

271. Hamid O, Robert C, Daud A, et al: Safety and tumor responses with lambrolizumab (anti-PD-1) in melanoma. N Engl J Med 369:134-44, 2013

272. Topalian SL, Hodi FS, Brahmer JR, et al: Safety, activity, and immune correlates of anti-PD-1 antibody in cancer, New England journal of medicine, 2012, pp 2443‐2454

273. Mo H, Huang J, Xu J, et al: Safety, anti-tumour activity, and pharmacokinetics of fixed-dose SHR-1210, an anti-PD-1 antibody in advanced solid tumours: a dose-escalation, phase 1 study. Br J Cancer 119:538-545, 2018

274. Spigel DR, McCleod M, Jotte RM, et al: Safety, Efficacy, and Patient-Reported Health-Related Quality of Life and Symptom Burden with Nivolumab in Patients with Advanced Nonâ€“Small Cell Lung Cancer, Including Patients Aged 70 Years or Older with Poor Performance Status (CheckMate 153), Journal of thoracic oncology, 2019

275. Bjoern J, Iversen TZ, Nitschke NJ, et al: Safety, immune and clinical responses in metastatic melanoma patients vaccinated with a long peptide derived from indoleamine 2,3-dioxygenase in combination with ipilimumab. Cytotherapy 18:1043-1055, 2016

276. Weber JS, Gibney G, Sullivan RJ, et al: Sequential administration of nivolumab and ipilimumab with a planned switch in patients with advanced melanoma (CheckMate 064): an open-label, randomised, phase 2 trial, The lancet. Oncology, 2016, pp 943‐955

277. Huang AC, Orlowski RJ, Xu X, et al: A single dose of neoadjuvant PD-1 blockade predicts clinical outcomes in resectable melanoma. BMC Cancer 25:454-461, 2019

278. Long GV, Atkinson V, Cebon JS, et al: Standard-dose pembrolizumab in combination with reduced-dose ipilimumab for patients with advanced melanoma (KEYNOTE-029): an open-label, phase 1b trial, The lancet. Oncology, 2017, pp 1202‐1210

279. Kato K, Satoh T, Muro K, et al: A subanalysis of Japanese patients in a randomized, double-blind, placebo-controlled, phase 3 trial of nivolumab for patients with advanced gastric or gastro-esophageal junction cancer refractory to, or intolerant of, at least two previous chemotherapy regimens (ONO-4538-12, ATTRACTION-2), Gastric cancer, 2019, pp 344‐354

280. Burger JA, Wiestner A, Wu CJ, et al: Surgical Outcomes After Neoadjuvant Chemotherapy and Ipilimumab for Non-Small Cell Lung Cancer. Nat Commun 105:924-929, 2018

281. Lebbé C, Weber JS, Maio M, et al: Survival follow-up and ipilimumab retreatment of patients with advanced melanoma who received ipilimumab in prior phase II studies, Annals of oncology : official journal of the european society for medical oncology, 2014, pp 2277‐2284

282. Ascierto PA, Long GV, Robert C, et al: Survival Outcomes in Patients with Previously Untreated BRAF Wild-Type Advanced Melanoma Treated with Nivolumab Therapy: three-Year Follow-up of a Randomized Phase 3 Trial, JAMA oncology, 2018

283. McDermott DF, Drake CG, Sznol M, et al: Survival, Durable Response, and Long-Term Safety in Patients With Previously Treated Advanced Renal Cell Carcinoma Receiving Nivolumab. J Clin Oncol 33:2013-20, 2015

284. Topalian SL, Sznol M, McDermott DF, et al: Survival, durable tumor remission, and long-term safety in patients with advanced melanoma receiving nivolumab. J Clin Oncol 32:1020-30, 2014

285. Chatterjee M, Turner DC, Felip E, et al: Systematic evaluation of pembrolizumab dosing in patients with advanced non-small-cell lung cancer, Annals of oncology : official journal of the european society for medical oncology, 2016, pp 1291‐1298

286. Puzanov I, Milhem MM, Minor D, et al: Talimogene Laherparepvec in Combination With Ipilimumab in Previously Untreated, Unresectable Stage IIIB-IV Melanoma. J Clin Oncol 34:2619-26, 2016

287. Tobin RP, Jordan KR, Robinson WA, et al: Targeting myeloid-derived suppressor cells using all-trans retinoic acid in melanoma patients treated with Ipilimumab, International immunopharmacology, 2018, pp 282‐291

288. Ready N, Farago AF, de Braud F, et al: Third-Line Nivolumab Monotherapy in Recurrent SCLC: checkMate 032, Journal of thoracic oncology, 2019, pp 237‐244

289. Di Giacomo AM, Ascierto PA, Queirolo P, et al: Three-year follow-up of advanced melanoma patients who received ipilimumab plus fotemustine in the Italian Network for Tumor Biotherapy (NIBIT)-M1 phase II study. Ann Oncol 26:798-803, 2015

290. Lyerly HK, Fujiwara Y: Tolerability and efficacy of durvalumab in Japanese patients with advanced solid tumors. J Thromb Thrombolysis 110:1715-1723, 2019

291. Escudier B, Motzer RJ, Sharma P, et al: Treatment Beyond Progression in Patients with Advanced Renal Cell Carcinoma Treated with Nivolumab in CheckMate 025, European urology, 2017, pp 368‐376

292. Maio M, Scherpereel A, Calabrò L, et al: Tremelimumab as second-line or third-line treatment in relapsed malignant mesothelioma (DETERMINE): a multicentre, international, randomised, double-blind, placebo-controlled phase 2b trial, The lancet. Oncology, 2017, pp 1261‐1273

293. Calabro L, Morra A, Giannarelli D, et al: Tremelimumab combined with durvalumab in patients with mesothelioma (NIBIT-MESO-1): an open-label, non-randomised, phase 2 study. Lancet Respir Med 6:451-460, 2018

294. Calabro L, Morra A, Fonsatti E, et al: Tremelimumab for patients with chemotherapy-resistant advanced malignant mesothelioma: an open-label, single-arm, phase 2 trial. Lancet Oncol 14:1104-1111, 2013

295. Vonderheide RH, LoRusso PM, Khalil M, et al: Tremelimumab in combination with exemestane in patients with advanced breast cancer and treatment-associated modulation of inducible costimulator expression on patient T cells. Clin Cancer Res 16:3485-94, 2010

296. Reck M, Rodriguez-Abreu D, Robinson AG, et al: Updated analysis of KEYNOTE-024: pembrolizumab versus platinum-based chemotherapy for advanced non–small-cell lung cancer with PD-L1 tumor proportion score of 50% or greater, Journal of clinical oncology, 2019, pp 537‐546

297. Fehrenbacher L, von Pawel J, Park K, et al: Updated Efficacy Analysis Including Secondary Population Results for OAK: a Randomized Phase III Study of Atezolizumab versus Docetaxel in Patients with Previously Treated Advanced Non-Small Cell Lung Cancer, Journal of thoracic oncology, 2018, pp 1156‐1170

298. Use of archival versus newly collected tumor samples for assessing PD-L1 expression and overall survival: an updated analysis of KEYNOTE-010 trial, Annals of oncology : official journal of the european society for medical oncology, 2019, pp 281‐289
